# Aortic valve stenosis serum IGFBP2 mediates female-specific Evogliptin resistance in valve myofibroblasts

**DOI:** 10.1016/j.celrep.2026.117678

**Published:** 2026-07-13

**Authors:** Brandon J. Vogt, Megan Chavez, Nicole E. Félix Vélez, Rayyan M. Gorashi, Ryan R. Reeves, Brian A. Aguado

**Affiliations:** 1 Shu Chien-Gene Lay Department of Bioengineering, University of California, San Diego, La Jolla, CA, USA; 2 Sanford Consortium for Regenerative Medicine, La Jolla, CA, USA; 3 Sulpizio Cardiovascular Center, University of California, San Diego, La Jolla, CA, USA; 4 Program in Materials Science and Engineering, University of California, San Diego, La Jolla, CA, USA; 5 Lead contact

## Abstract

Aortic valve stenosis (AVS) is a sexually dimorphic cardiovascular disease characterized by fibro-calcification of the aortic valve leaflet. Sex differences in AVS arise in part from sexually dimorphic serum composition that differentially regulates valvular interstitial cell (VIC) myofibroblast activation. However, how individual serum factors contribute to sex-specific drug responses remains unknown. Here, we integrate serum proteomic profiling with *in vitro* drug screening using hydrogel biomaterials to identify sex-specific regulators of antifibrotic drug efficacy. We found that serum insulin-like growth factor binding protein 2 (IGFBP2) mediates Evogliptin resistance in female VICs cultured with female AVS serum through the activation of Rho/ROCK and focal adhesion kinase signaling. Our findings highlight IGFBP2 as a candidate biomarker for stratifying female patients with AVS for Evogliptin treatment, underscoring the need to incorporate sex as a biological variable in determining AVS treatments.

## In brief

Vogt et al. show that serum IGFBP2 levels predict the anti-fibrotic response to Evogliptin in female valvular myofibroblasts. Acknowledging that aortic valve stenosis progresses differently between sexes, determining sex-dependent biomarkers that cause drug resistance offers paths for improved precision treatments for patients with early-stage aortic valve stenosis.

## Graphical Abstract

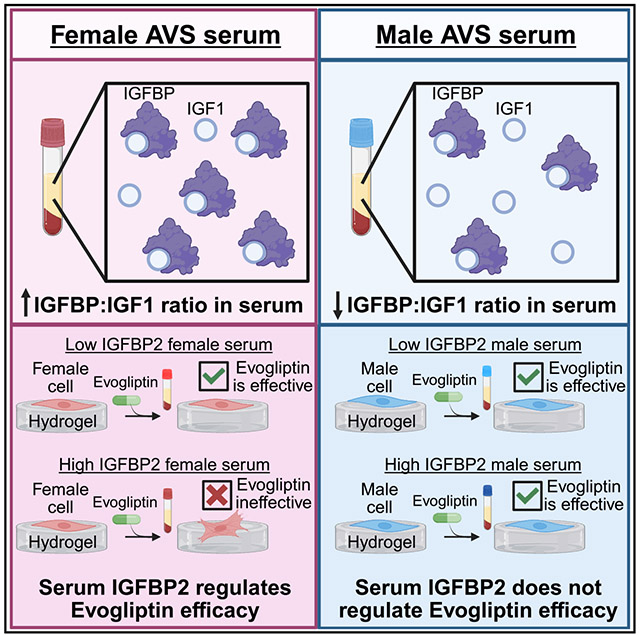

## INTRODUCTION

Aortic valve stenosis (AVS) is characterized by extensive fibrosis and calcification of the aortic valve leaflet that impedes cardiovascular function.^1,2^ As AVS develops, patients experience impaired hemodynamics, left ventricular pressure overload, and eventual heart failure.^3^ AVS risk increases dramatically with age and is highly prevalent in elderly populations, with approximately 1 in 8 adults over the age of 75 affected.^4^ Moreover, population aging is expected to drive increases in both AVS incidence and treatment costs, with an estimated 19 million individuals expected to be affected by 2036 and costs projected to reach $19 billion by 2034.^5-7^ Currently, AVS is treated through interventional procedures such as surgical or transcatheter aortic valve replacements which implant a new valve to restore normal hemodynamic function in the heart and prolong patient survival.^8,9^ However, these interventions pose their own procedural risks and only serve as temporary solutions, as patients can experience failure of the newly implanted valve.^10,11^

To address these limitations, researchers and clinicians have long pursued a variety of pharmacologic strategies to slow or halt AVS progression, resulting in multiple clinical trials.^12-16^ Statins are the most extensively investigated drugs for AVS, owing to their proven efficacy in atherosclerosis, which involves similar pathogenic processes such as lipid accumulation and oxidation.^17,18^ However, large-scale trials of statins and other lipid-lowering drugs have yielded little to no success in halting AVS progression.^14-16^ Other drug classes, including anti-inflammatory drugs and bisphosphonates, have also been explored with similar disappointing outcomes.^12,13^ More recently, inhibitors such as Evogliptin have shown early promise in suppressing valvular calcification.^19-21^ However, despite these efforts, current therapeutic options remain largely limited to symptom management, including antihypertensive and renin-angiotensin-aldosterone system-targeting drugs to reduce cardiac load.^22^ Given there are still no approved drugs to reverse AVS progression, there is an active need for patient-specific treatment strategies.

AVS is a heterogeneous disease with significant patient-to-patient variability and a wide range of disease phenotypes, likely necessitating a precision medicine approach for identifying effective drug treatments.^23-26^ Specifically, AVS progression is sex-dependent, with male patients with AVS experiencing elevated calcification of the aortic valve relative to female patients with AVS, who instead show increased fibrosis at the early stages of disease.^27-29^ Observed sex differences in AVS progression extend to clinical treatment responses, such as increased mortality following surgical valve replacement in female patients and increased mortality following transcatheter valve replacement in male patients.^30-32^ Sex-based disparities are further compounded by the fact that female patients with AVS are more often underdiagnosed and are referred for intervention at later disease stages than men.^33-36^ As a result, researchers and clinicians have developed sex-specific risk profiles for the recommendation of valve replacement procedures.^37,38^

Interestingly, sex differences in AVS have also been linked to the biological sex of aortic valve cells. Male and female valvular interstitial cells (VICs), the resident fibroblast of the aortic valve, show divergent phenotypes in response to mechanical and biochemical cues.^39,40^ These sex differences are best captured on hydrogel biomaterials used as cell culture platforms that recapitulate the stiffness of aortic valve tissue.^41-43^ Specifically, female VICs with XX chromosomes cultured on hydrogels show elevated myofibroblast activation, a pro-fibrotic phenotype associated with extracellular matrix turnover and collagen deposition.^39,44^ In contrast, male VICs with XY chromosomes cultured on hydrogels are more prone to differentiating into an osteoblast-like phenotype that promotes the calcification of the aortic valve.^45,46^ Cellular scale sex differences also regulate drug responses, with recent findings showing that sex chromosome-linked genes contribute to sex-specific drug efficacy in VICs.^39^ These differences have been leveraged to develop sex-specific predictive models of VIC drug responses and identify sex-biased drug treatment parameters to improve drug responses in both male and female VICs.^47^ Collectively, these findings suggest that sexually dimorphic VIC phenotypes play a central role in AVS progression and may contribute to variations in clinical treatment outcomes.

Inflammatory factors present in AVS serum have also been shown to promote pathological VIC phenotypes.^42,48^ AVS serum contains a myriad of cytokines, growth factors, and extracellular matrix components that can individually and collectively interact to alter VIC function.^49,50^ Notably, following transcatheter aortic valve replacement, the serum proteome undergoes significant changes that are broadly associated with maintaining VIC quiescence.^51^ Interestingly, serum samples from male and female patients show enrichment of distinct clusters associated with AVS progression, such as increased actin binding in female serum and increased cholesterol metabolism in male serum.^52^ Moreover, patient-specific serum proteomic profiles have been used to accurately predict VIC drug responses.^53^ These findings suggest that the serum proteome contributes to patient heterogeneity, individualized drug responses, and sex-specific mechanisms of AVS development. However, the mechanisms by which individual serum factors influence VIC drug responses in a patient- and sex-specific manner remain unknown.

In this study, we utilized hydrogel biomaterials and serum proteomic analyses to identify predictive links between serum factor abundance and myofibroblast responses to antifibrotic drugs *in vitro*. We hypothesized that individual serum biomarkers would be predictive of drug responses in VICs and that male and female serum contain distinct biomarkers that contribute to patient-specific drug sensitivity. To test these hypotheses, we cultured male and female VICs with sex-matched AVS patient serum and treated them with one of the three antifibrotic drugs that are in ongoing clinical trials for treating AVS: Ataciguat (NCT07001800), Evogliptin (NCT05143177), or Colchicine (NCT05162742).^19-21^ By correlating *in vitro* drug responses with serum protein abundance, we identified a female-specific association between elevated insulin-like growth factor binding protein 2 (IGFBP2) levels and reduced efficacy of the dipeptidylpeptidase 4 inhibitor, Evogliptin. Additionally, we found that female serum IGFBP2 levels regulate Evogliptin sensitivity in VICs through Rho/ROCK and focal adhesion kinase (FAK) signaling pathways. These findings were confirmed through neutralizing antibody experiments to block the effects of IGFBP2 and inhibiting Rho/ROCK and FAK signaling to restore Evogliptin efficacy in female VICs cultured with female serum. Together, these findings highlight IGFBP2 as a key mediator of Evogliptin resistance in female VICs and a potential biomarker for patient stratification in ongoing clinical trials.

## RESULTS

### Proteomic profiling reveals distinct serum signatures in patients with AVS

**Figure 1.**
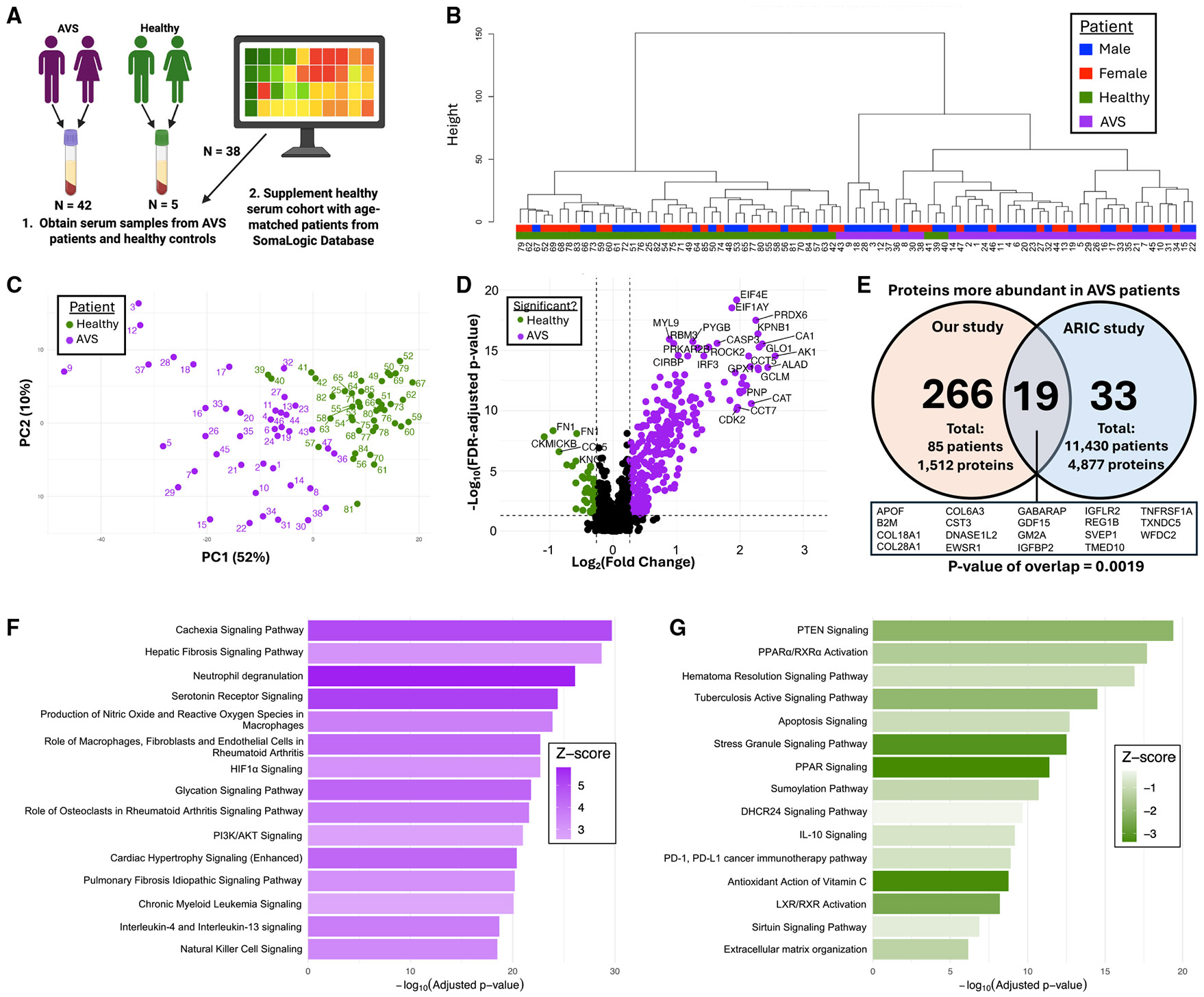
Proteomic profiling reveals distinct serum signatures in patients with AVS (A) Schematic overview of the approach to obtain and supplement serum samples for proteomic analysis. Figure created in BioRender: Aguado, B. (2026) https://BioRender.com/g4omlx4. (B) Hierarchical clustering dendrogram links all serum samples based on proteomic data (*N* = 43 healthy, *N* = 42 AVS). (C) Principal component analysis of the serum proteome from healthy and patients with AVS (*N* = 43 healthy, *N* = 42 AVS). (D) Volcano plot of 1,512 protein abundances enriched in healthy serum samples (green) or AVS serum samples (purple) (*N* = 43 healthy, *N* = 42 AVS). Statistical significance was defined as false-discovery rate (FDR) adjusted *p* value <0.05 (unpaired *t* test with Welch’s correction) and fold change greater than 1.2. (E) Venn diagram compares serum biomarkers identified in this study against the serum biomarkers identified in the Atherosclerosis Risk In Communities study.^56^ Statistical significance was determined by Fisher’s exact test. (F and G) Ingenuity pathway analysis shows the top pathways associated with proteins that are more abundant in (F) AVS serum samples and (G) healthy serum samples.

We first sought to identify proteins that are upregulated in AVS patient serum relative to healthy controls. Based on echocardiographic and patient data, the male AVS cohort was younger and had a 1.5-fold larger aortic valve area relative to the female AVS cohort (Table S1). All serum samples were analyzed using the SomaLogic DNA aptamer-based proteomic platform,^54,55^ which quantified the relative abundance of a pre-selected panel of 1,512 proteins with known associations with inflammation or cardiovascular disease. To increase the size of our healthy control group, we supplemented our cohort with 38 age-matched individuals from the SomaLogic serum proteome database (Figure 1A). Unsupervised hierarchical clustering revealed that AVS and healthy serum samples largely cluster together, suggesting significant proteomic changes occur during AVS progression (Figure 1B). Principal component analysis confirmed that healthy serum samples cluster separately from AVS serum and that AVS serum exhibits markedly greater heterogeneity (Figure 1C).

We next performed a differential protein analysis and identified 41 proteins more abundant in healthy serum and 285 proteins more abundant in AVS serum (Figure 1D). A similar comparison between male and female AVS serum revealed minimal baseline sex differences, with only five proteins more abundant in female AVS serum, and none more abundant in male AVS serum (Figure S1). To further narrow down candidate AVS biomarkers, we compared our identified proteins with AVS biomarkers identified from the Atherosclerosis Risk In Communities study that tracked over 11,000 patients over three decades.^56^ We found a significant overlap between the two datasets, corroborating our findings and narrowing down our biomarker pool to 19 robustly validated serum proteins (Figure 1E). Using all 285 serum factors more abundant in AVS patient serum, we used ingenuity pathway analysis (IPA) to identify pathways altered during AVS progression. The top enriched pathways were linked to fibrotic and cardiovascular disease processes, inflammatory signaling, and altered immune cell function (Figure 1F). Complementary analysis using the Kyoto Encyclopedia of Genes and Genomes (KEGG) database revealed similar results, with the top pathways linked to inflammation and cardiovascular disease (Figure S2). We also used IPA on proteins more abundant in healthy serum and observed enrichment of anti-inflammatory and metabolic pathways (Figure 1G). However, KEGG analysis did not identify any significantly enriched pathways, likely due to the limited number of proteins upregulated in the healthy cohort.

### Clinically relevant doses of ataciguat, colchicine, and evogliptin reduce myofibroblast activation in male and female VICs

**Figure 2.**
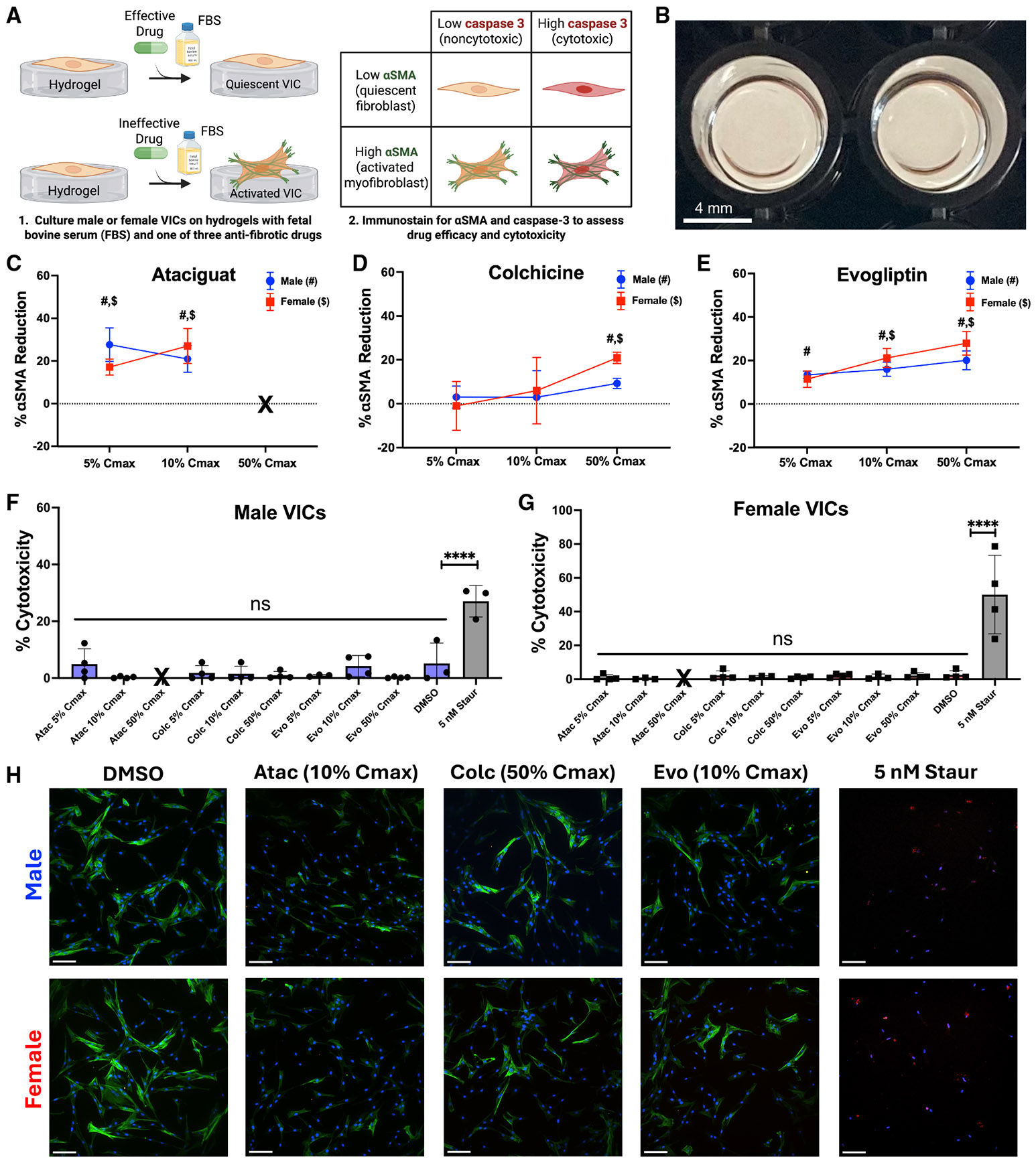
Clinically relevant doses of antifibrotic drugs prevent male and female VIC myofibroblast activation (A) Schematic overview of the protocol to optimize antifibrotic drug dosing using hydrogel biomaterials. Figure created in BioRender. Aguado, B. (2026) https://BioRender.com/lue0gxl. (B) Representative image of formed hydrogels ready for cell seeding. (C–E) Percent αSMA reduction in male and female VICs cultured on hydrogels with increasing Cmax concentrations of (C) Ataciguat, (D) Colchicine, or (E) Evogliptin (*N* = 3–4 gels). X indicates complete cell death at that concentration. Statistical significance is indicated as # = *p* < 0.05 for male VICs and $ = *p* < 0.05 for female VICs (one-sample t and Wilcoxon tests against a theoretical mean of zero). Data are shown as mean ± standard error of the mean. (F and G) Percent cytotoxicity in (F) male and (G) female VICs in response to increasing Cmax concentrations of Ataciguat, Colchicine, or Evogliptin (N = 3–4 gels). A 5 nM dose of Staurosporine was used as a positive control for cell death. Statistical significance is indicated as **** = *p* < 0.0001 (one-way ANOVA with Tukey posttests). (H) Representative immunofluorescent images of male and female VICs cultured on hydrogels with different experimental conditions (green: αSMA, blue: nuclei, red: cleaved caspase-3). Scale bars, 100 μm. Abbreviations: Atac = Ataciguat, Colc = Colchicine, Evo = Evogliptin, Staur = Staurosporine.

Next, we optimized doses of three antifibrotic drugs currently in clinical trials for treating AVS (Ataciguat, Colchicine, and Evogliptin).^19-21^ Male or female VICs were treated with fetal bovine serum (FBS) and either 5%, 10%, or 50% of each drug’s maximum serum concentration (Cmax) (Table S2). After two days of treatment, VICs were immunostained for alpha smooth muscle actin (αSMA) and cleaved caspase-3 to assess myofibroblast activation and cell viability (Figure 2A). All experiments were performed using our previously optimized poly(ethylene glycol) hydrogel formulation with an elastic modulus of ~51 kPa (Figure 2B).^57^ This platform mirrors the stiffness of diseased aortic valve tissue and provides a physiologically relevant cell culture environment known to influence sex-specific myofibroblast activation and drug sensitivity.^47^

Using these hydrogels, we found that Ataciguat had a maximal efficacy at a 10% Cmax dose, as indicated by the greatest reduction in αSMA gradient mean intensity relative to vehicle control (Figure 2C). Ataciguat at a 50% Cmax dose was fully cytotoxic and not quantified. Colchicine and Evogliptin were similarly effective in male and female VICs at 50% and 10% Cmax doses, respectively (Figures 2D and 2E). No sex differences in drug efficacy were observed across any dosing parameters tested when using fetal bovine serum. Importantly, all optimized drug doses were confirmed to have minimal cytotoxicity in both male and female VICs, suggesting that optimized drug concentrations are both efficacious and well-tolerated (Figures 2F-2H).

### Sex-specific serum biomarkers predict antifibrotic drug efficacy

**Figure 3.**
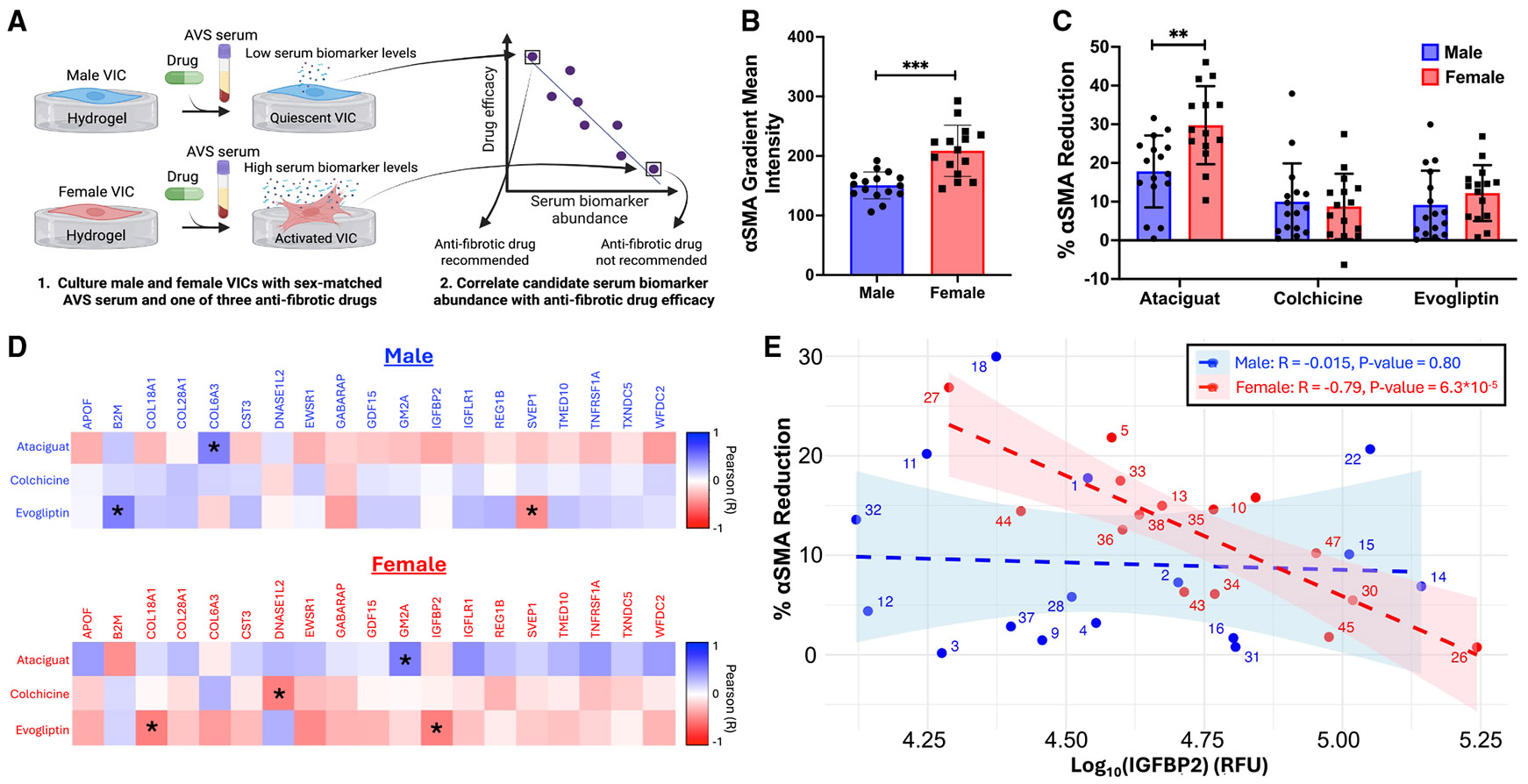
Sex-specific serum biomarkers regulate antifibrotic drug efficacy (A) Schematic overview of the approach to link candidate serum biomarkers with *in vitro* drug efficacy. Figure created in BioRender. Aguado, B. (2026) https://BioRender.com/9cyhk4j. (B) αSMA gradient mean intensity for male and female VICs cultured on hydrogels with individual sex-matched AVS patient serum samples (*N* = 16 male serum samples, *N* = 15 female serum samples). Statistical significance is indicated as *** = *p* < 0.001 (unpaired two-tailed *t* test with Welch’s correction). (C) Percent αSMA reduction in male and female VICs cultured on hydrogels with optimized doses of Ataciguat, Colchicine, or Evogliptin (*N* = 16 male serum samples, *N* = 15 female serum samples). Statistical significance is indicated as ** = *p* < 0.01 (unpaired two-tailed *t* test with Welch’s correction). (D) Heatmaps show the correlation between each candidate serum factor and antifibrotic drug efficacy for male or female VICs cultured with sex-matched AVS serum (*N* = 16 male, *N* = 15 female). Statistical significance is indicated as * = *p* < 0.05 (paired two-tailed *t* test). (E) Correlation between serum IGFBP2 abundance (relative fluorescence units (RFU)) and percent αSMA reduction in male and female VICs cultured on gels with sex-matched AVS serum and Evogliptin (*N* = 16 male, *N* = 15 female).

We next sought to determine biomarkers in human serum that regulate antifibrotic drug efficacy *in vitro*. To test this, male or female VICs were seeded on hydrogels and treated with sex-matched serum and optimized doses of Ataciguat, Colchicine, or Evogliptin. After two days, VICs were fixed and immunostained for αSMA to quantify myofibroblast responses to each drug. For each serum sample, drug efficacy was plotted against the relative abundance of each of the 19 candidate biomarkers to generate robust, sex-specific correlations (Figure 3A). At baseline, female VICs cultured with female AVS serum exhibited a 1.4-fold higher rate of αSMA expression relative to male VICs cultured with male AVS serum (Figure 3B). Additionally, Ataciguat demonstrated a 1.6-fold greater efficacy in reducing myofibroblast activation in female VICs relative to male VICs, whereas Colchicine and Evogliptin were similarly effective in both sexes (Figure 3C).

Overall correlations between drug efficacy and serum factor abundance were first assessed by pooling male and female samples (Table S3). Across all samples, we identified three significant correlations between serum protein abundance and Evogliptin efficacy, whereas no significant correlations were observed with Ataciguat or Colchicine (Figure S3). Sex-specific analyses revealed that correlations between drug efficacy and serum biomarker abundance differed significantly between male and female samples (Tables S4 and S5). More specifically, female VICs cultured with female AVS serum exhibited predominantly positive correlations between serum biomarker abundance and Ataciguat efficacy, and predominantly negative correlations for Colchicine or Evogliptin. In contrast, male VICs cultured with male AVS serum displayed the opposite trend, with serum factor abundance negatively correlating with Ataciguat efficacy and positively correlating with Colchicine and Evogliptin efficacy (Figure S4).

Next, we determined male-specific and female-specific correlations between drug efficacy and individual serum factor abundance. In male VICs cultured with male AVS serum, Collagen alpha-3(VI) chain (COL6A3) correlated positively with Ataciguat response (Pearson’s R = 0.495), and Beta-2-microglobulin (B2M) correlated positively with Evogliptin (Pearson’s R = 0.497), whereas Sushi, von Willebrand factor type A, epidermal growth factor, and pentraxin domain containing 1 (SVEP1) showed a significant negative correlation with Evogliptin (Pearson’s R = −0.444) (Figure 3D). In contrast, in female VICs cultured with female AVS serum, Ganglioside GM2 activator (GM2A) was positively correlated with Ataciguat efficacy (Pearson’s R = 0.510), while Deoxyribonuclease-1-like 2 (DNASE1L2) was negatively correlated with Colchicine efficacy (Pearson’s R = −0.531). Additionally, Collagen alpha-1(XVIII) chain (COL18A1) and Insulin-like growth factor binding protein 2 (IGFBP2) were negatively correlated with Evogliptin efficacy (Pearson’s R = −0.613, Pearson’s R = −0.791). Of these correlations, the strongest sex-specific association was the female-specific negative correlation between serum IGFBP2 abundance and Evogliptin efficacy (Figure 3E). Interestingly, male serum IGFBP2 levels did not correlate with Evogliptin response, suggesting that IGFBP2 may uniquely regulate Evogliptin sensitivity in female VICs cultured with female AVS serum.

### IGFBP2 promotes evogliptin resistance only in female VICs cultured with female AVS serum

**Figure 4.**
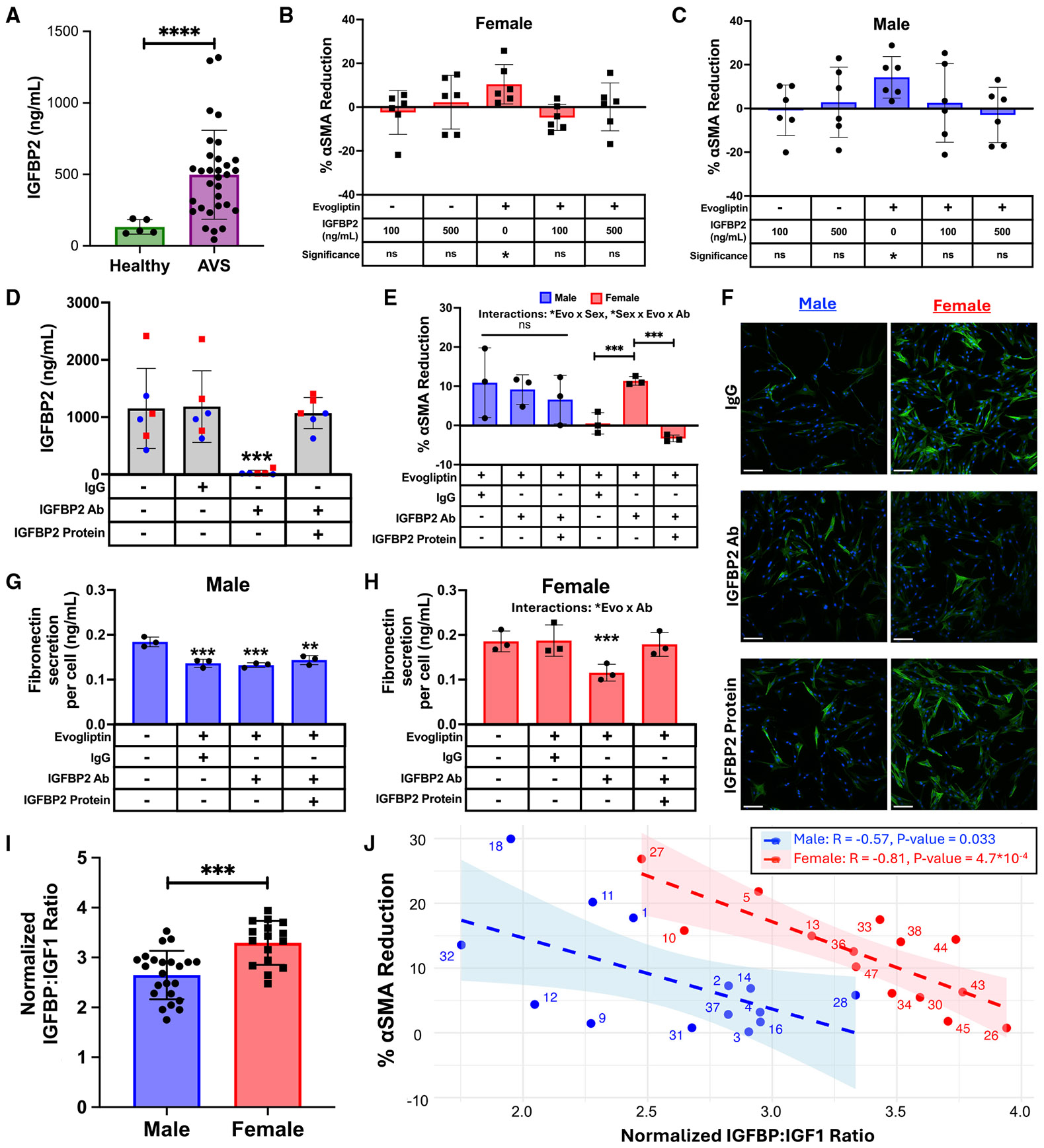
IGFBP2 mediates Evogliptin resistance in female VICs cultured with female AVS serum (A) Serum IGFBP2 concentrations quantified using an ELISA kit (*N* = 31 AVS, *N* = 5 healthy). Statistical significance is indicated as **** = *p* < 0.0001 (unpaired two-tailed *t* test with Welch’s correction). (B and C) Percent αSMA reduction in (B) female and (C) male VICs cultured on hydrogels with FBS and treated with Evogliptin and/or recombinant human IGFBP2 protein (*N* = 6 gels). Statistical significance is indicated as * = *p* < 0.05 (one-sample t and Wilcoxon test against a theoretical mean of zero). (D) Serum IGFBP2 levels quantified using an ELISA kit with and without IGFBP2 neutralizing antibody (IGFBP2 Ab), IgG control antibody, and recombinant human IGFBP2 protein (*N* = 3 male, *N* = 3 female). Statistical significance is indicated as *** = *p* < 0.001 (one-way ANOVA with Tukey posttests). (E) Percent αSMA reduction in male and female VICs cultured on hydrogels with sex-matched AVS patient serum samples, Evogliptin, IgG, IGFBP2 Ab, or recombinant human IGFBP2 protein (*N* = 3 male, *N* = 3 female). Statistical significance between groups is indicated as *** = *p* < 0.001, and significant interactions between variables are indicated as * = *p* < 0.05 (three-way ANOVA with Tukey posttests). Abbreviations: Evo = Evogliptin, Ab = IGFBP2 antibody. (F) Representative immunofluorescent images of male and female VICs cultured on hydrogels (green: αSMA, blue: nuclei). Scale bars, 100 μm. (G and H) Fibronectin secretion from cell culture supernatant collected from (G) male and (H) female VICs after two days of culture on hydrogels with sex-matched AVS patient serum samples, Evogliptin, IgG, IGFBP2 Ab, or recombinant human IGFBP2 (*N* = 3 male, *N* = 3 female). Statistical significance between groups relative to vehicle control is indicated as ** = *p* < 0.01, *** = *p* < 0.001, and significant interactions between variables are indicated as * = *p* < 0.05 (two-way ANOVA with Tukey posttests). (I) Normalized IGFBP to IGF1 ratio for male and female AVS serum samples (*N* = 23 male, *N* = 16 female). Statistical significance is indicated as *** = *p* < 0.001 (unpaired two-tailed *t* test with Welch’s correction). (J) Correlation between normalized IGFBP to IGF1 ratios and percent αSMA reduction in male and female VICs cultured on hydrogels with sex-matched serum and Evogliptin (*N* = 14 male, *N* = 14 female).

We next tested whether IGFBP2 can directly modulate female VIC responses to Evogliptin. First, we observed that IGFBP2 is more abundant in AVS serum relative to healthy controls as quantified via SomaScan array (Figure S5). We also detected a near 4-fold increase in IGFBP2 concentrations in AVS serum relative to healthy serum samples as quantified via enzyme-linked immunosorbent assay (ELISA) (Figure 4A). A strong correlation between measured IGFBP2 serum concentration and IGFBP2 serum abundance was observed (Pearson’s R = 0.921), indicating accurate serum measurements from both assays (Figure S6). IGFBP2 concentrations were similar between male and female AVS serum samples (Figure S7).

Using IGFBP2 doses corresponding to measured healthy and AVS serum levels, we next assessed the effects of IGFBP2 on Evogliptin efficacy. Female VICs cultured on hydrogels with FBS showed no baseline response to 100 or 500 ng/mL IGFBP2. However, the ability of Evogliptin to reduce myofibroblast activation was abolished in the presence of 100 or 500 ng/mL IGFBP2, confirming that physiologically relevant concentrations of IGFBP2 can regulate Evogliptin efficacy in female VICs (Figure 4B). Repeating this experiment with male VICs yielded similar results, suggesting that the female-specific correlation between IGFBP2 serum levels and Evogliptin efficacy is dependent on differences in serum composition rather than intrinsic male vs. female VIC drug sensitivity (Figure 4C).

To test this hypothesis, we next aimed to neutralize IGFBP2 in serum using an antibody and rescue IGFBP2 effects by adding excess recombinant human IGFBP2 protein. We selected the three male and female AVS serum samples with the highest IGFBP2 levels and confirmed that the neutralizing antibody effectively blocked IGFBP2 (Figure 4D). IGFBP2 stability in serum over the two-day culture period was also verified to ensure minimal degradation (Figure S8). Male or female VICs were then seeded on hydrogels and cultured with Evogliptin under three conditions: (1) sex-matched serum with a control antibody, (2) sex-matched serum with IGFBP2 neutralized, or (3) sex-matched serum with excess IGFBP2 added after neutralization. Male VICs responded to Evogliptin at baseline and showed no changes in Evogliptin response with IGFBP2 neutralization or rescue (Figures 4E and 4F). In contrast, female VICs were initially unresponsive to Evogliptin but became responsive when IGFBP2 was neutralized. Restoring IGFBP2 protein levels again negated the effects of Evogliptin, demonstrating that IGFBP2 mediates Evogliptin resistance specifically in female VICs cultured with female AVS serum. These results were confirmed by collecting the cell culture supernatant and measuring the levels of secreted fibronectin in each condition as an additional assay to assess myofibroblast deactivation (Figures 4G and 4H). Specifically, male VICs showed reduced fibronectin secretion in response to Evogliptin regardless of IGFBP2 neutralization or restoration, whereas female VICs showed reduced fibronectin secretion in response to Evogliptin only with IGFBP2 neutralization.

Given the unique female response to IGFBP2 neutralization, we next investigated whether overall insulin-like growth factor 1 (IGF1) signaling is altered in female AVS serum. We calculated the normalized insulin-like growth factor binding protein (IGFBP) to IGF1 ratio in male and female AVS serum and found that female serum exhibited a 1.25-fold higher ratio, suggesting a relative reduction in factors promoting IGF1 signaling (Figure 4I). To confirm this difference was not due to age differences between male and female patients with AVS, we correlated patient age with serum IGFBP2 abundance, serum IGF1 abundance, and the normalized IGFBP:IGF1 ratio (Figure S9). We found no significant correlations between male or female AVS patient age and these factors. However, patient age did strongly correlate with serum IGFBP2 abundance, serum IGF1 abundance, and normalized IGFBP:IGF1 ratio in healthy male and female patients, apart from IGF1 in the healthy female group. Correlating the normalized IGFBP:IGF1 ratio with Evogliptin efficacy in VICs cultured with sex-matched AVS serum revealed a significant negative correlation in both male and female VICs (Figure 4J). Together, these data indicate that an elevated IGFBP to IGF1 ratio contributes to reduced Evogliptin efficacy and that female serum is particularly susceptible to IGFBP2-mediated Evogliptin resistance due to this elevated ratio.

### RNA sequencing shows that IGFBP2 acts through mechanosensing pathways to promote evogliptin resistance in female VICs

**Figure 5.**
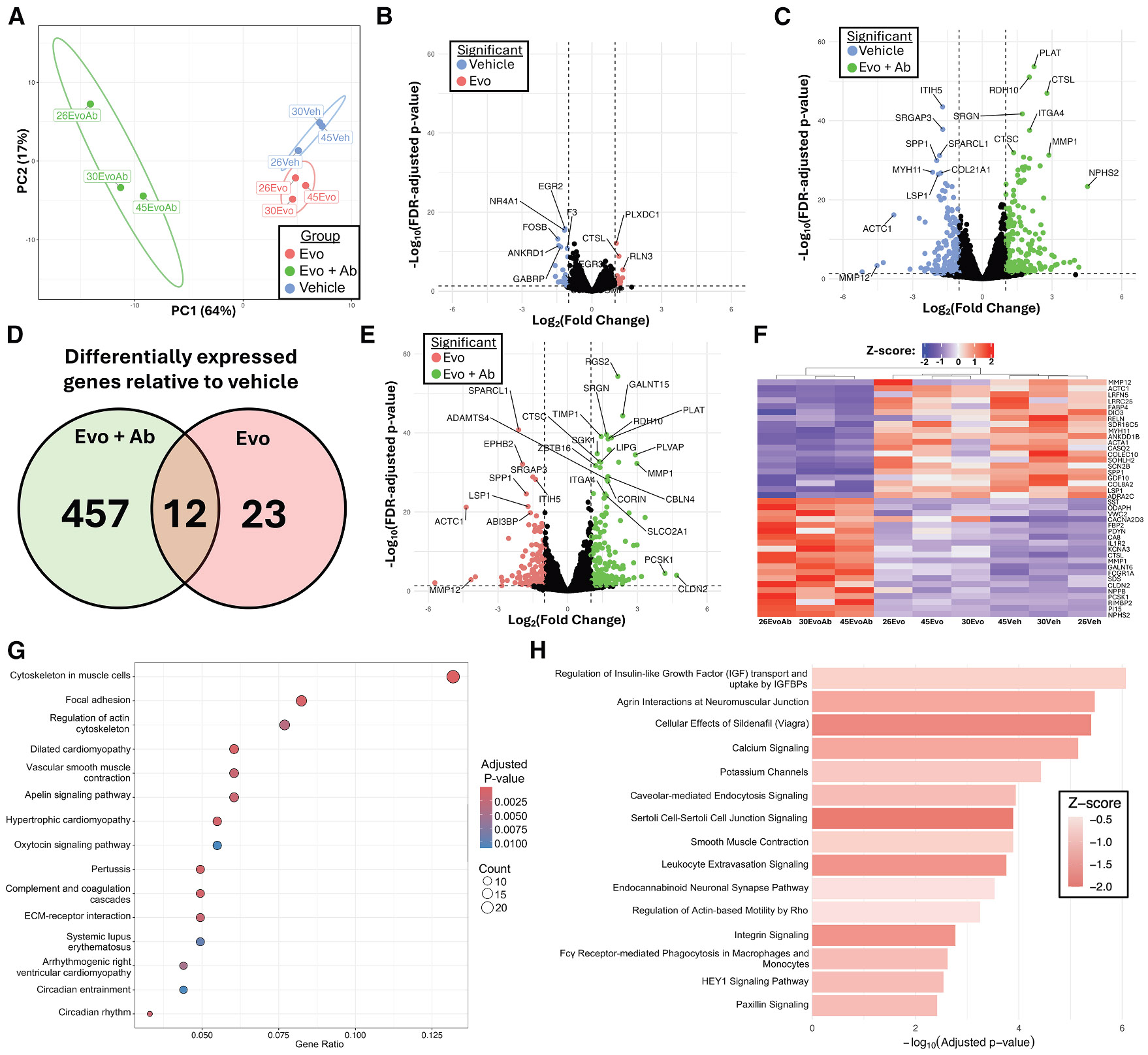
RNA sequencing shows that IGFBP2 acts through mechanosensing pathways to promote Evogliptin resistance in female VICs (A) Principal component analysis clustering samples based on RNA sequencing results (*N* = 3). (B and C) Volcano plot of differential gene expression from RNA sequencing between (B) Vehicle and Evogliptin groups or (C) Vehicle and Evogliptin plus IGFBP2 neutralizing antibody groups (*N* = 3). Statistical significance was defined as FDR-adjusted *p* value <0.05 (unpaired two-tailed *t* test with Welch’s correction) and fold change greater than 2. (D) Venn diagram compares differentially expressed genes in the Evogliptin and Evogliptin plus IGFBP2 neutralizing antibody groups relative to vehicle control (*N* = 3). (E) Volcano plot of differential gene expression from RNA sequencing between Evogliptin and Evogliptin plus IGFBP2 neutralizing antibody groups (*N* = 3). Statistical significance was defined as FDR-adjusted *p* value <0.05 (unpaired *t* test with Welch’s correction) and fold change greater than 2. (F) Heatmap of the top 20 up- and down-regulated genes in the Evogliptin plus IGFBP2 neutralizing antibody group relative to vehicle control (*N* = 3). (G and H) Top 15 upregulated pathways in Evogliptin versus Evogliptin plus IGFBP2 neutralizing antibody group identified by (G) KEGG and (H) Ingenuity Pathway Analysis. Abbreviations: Evo: Evogliptin, Evo + Ab: Evogliptin with IGFBP2 neutralizing antibody, Veh: Vehicle control. Number indicates the patient serum sample ID (26, 30, and 45).

Next, we performed RNA sequencing to investigate the pathways by which IGFBP2 promotes resistance to Evogliptin in female VICs cultured with female AVS serum. We profiled the transcriptomes of female VICs cultured under three conditions: (1) female AVS serum alone (vehicle), (2) female AVS serum with Evogliptin (Evo), and (3) female AVS serum with Evogliptin plus an IGFBP2-neutralizing antibody (EvoAb). As expected, IGFBP2 neutralization enhanced Evogliptin efficacy, as indicated by reduced gene expression of the myofibroblast marker, *ACTA2* (Figure S10). Principal component analysis demonstrated that the vehicle and Evo groups clustered closely together, whereas the EvoAb group was distinctly separated (Figure 5A). Differential gene expression analysis further supported this observation, with only 35 genes found to be significantly altered between the vehicle and Evo groups (Figure 5B). In contrast, 469 differentially expressed genes were detected between the vehicle and EvoAb groups (Figure 5C). These results indicate that IGFBP2 neutralization induces a robust transcriptomic shift during Evogliptin treatment. A comparison of differentially expressed genes relative to the vehicle group revealed 12 genes commonly enriched in both the Evo and EvoAb conditions (Figure 5D). Directly comparing the Evo and EvoAb groups resulted in 386 genes differentially expressed, with 182 genes upregulated in the Evo group (Figure 5E). Analyzing the 20 genes most upregulated or downregulated in the EvoAb group relative to the vehicle showed that the Evo group aligned closely with the vehicle group, further confirming the inefficacy of Evogliptin treatment in female VICs cultured with female AVS serum containing high levels of IGFBP2 (Figure 5F).

We next performed a pathway enrichment analysis to identify candidate signaling networks through which IGFBP2 promotes resistance to Evogliptin. Our KEGG pathway analysis on genes upregulated in the Evo group relative to the EvoAb group revealed enrichment of pathways related to mechanosensing, focal adhesion formation, and cytoskeletal regulation (Figure 5G). We also performed IPA and identified “Regulation of insulin-like growth factor (IGF) transport and uptake by IGFBPs” as the top pathway, confirming IGFBP2 neutralization in the EvoAb condition. Additional pathways enriched in the Evo group included Rho/ROCK signaling and integrin signaling (Figure 5H). In contrast, the top pathways enriched in the EvoAb group relative to the Evo group were primarily associated with biochemical signaling processes (Figure S11). Collectively, these analyses suggest that IGFBP2 may promote Evogliptin resistance by enhancing mechanosensing and downstream integrin-mediated signaling in female VICs.

### IGFBP2 drives female-specific evogliptin resistance through Rho/ROCK and focal adhesion kinase signaling

**Figure 6.**
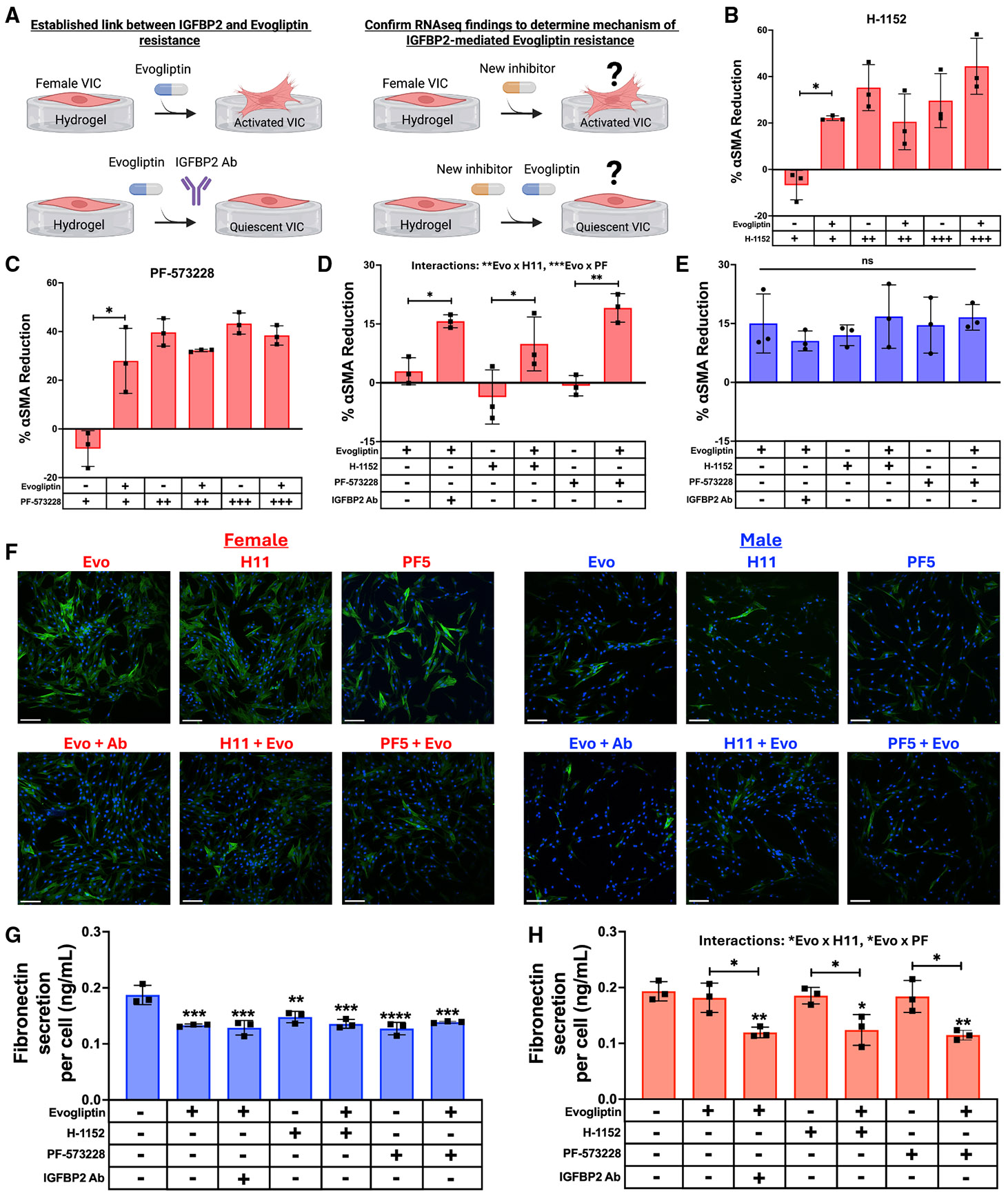
IGFBP2 drives female-specific Evogliptin resistance through Rho/ROCK and focal adhesion kinase signaling (A) Schematic overview of the approach to validate the mechanism of IGFBP2-mediated Evogliptin resistance. Figure Created in BioRender. Aguado, B. (2026) https://BioRender.com/2c3itp7. (B) Percent αSMA reduction in female VICs cultured on hydrogels with female AVS serum and treated with various doses of H-1152 with and without Evogliptin (*N* = 3 gels). For H-1152: + = 0.1 μM; ++ = 0.5 μM; +++ = 5 μM. Statistical significance is indicated as * = *p* < 0.05 (unpaired two-tailed *t* test with Welch’s correction). (C) Percent αSMA reduction in female VICs cultured on hydrogels with AVS serum and treated with various doses of PF-573228 with and without Evogliptin (*N* = 3 gels). For PF-573228: + = 0.1 μM; ++ = 1 μM; +++ = 10 μM. Statistical significance is indicated as * = *p* < 0.05 (unpaired two-tailed *t* test with Welch’s correction). (D and E) Percent αSMA reduction in (D) female or (E) male VICs cultured on hydrogels with sex-matched AVS serum and treated with various combinations of Evogliptin, IGFBP2 neutralizing antibody, H-1152, and PF-573228 (*N* = 3 serum samples per sex). Statistical significance between groups is indicated as * = *p* < 0.05, ** = *p* < 0.01, and significant interactions between variables are indicated as ** = *p* < 0.01, *** = *p* < 0.001 (two-way ANOVA with Tukey posttests). (F) Representative immunofluorescent images of male and female VICs cultured on hydrogels with sex-matched serum and various drug combinations (green: αSMA, blue: nuclei). Scale bars, 100 μm. Abbreviations: Evo = Evogliptin, H11 = H-1152, PF5 = PF-573228, Ab = IGFBP2 neutralizing antibody. (G and H) Fibronectin secretion from cell culture supernatant collected from (G) male and (H) female VICs after two days of culture on hydrogels with sex-matched AVS patient serum samples, Evogliptin, IGFBP2 Ab, H-1152, or PF-573228 (*N* = 3 male, *N* = 3 female). Statistical significance between groups or between groups relative to vehicle control is indicated as * = *p* < 0.05, ** = *p* < 0.01, *** = *p* < 0.001, **** = *p* < 0.0001, and significant interactions between variables are indicated as * = *p* < 0.05 (two-way ANOVA with Tukey posttests). Abbreviations: Evo = Evogliptin, H11 = H-1152, PF = PF-573228.

As a strategy to validate pathways identified through RNA sequencing, we next hypothesized that inhibiting mechanosensing pathways would sensitize female myofibroblasts to Evogliptin. Female VICs were cultured on hydrogels with female AVS patient serum and treated with inhibitors targeting Rho/ROCK or focal adhesion kinase (FAK) signaling in the presence and absence of Evogliptin (Figure 6A). Three doses of the Rho/ROCK inhibitor (H-1152) and the FAK inhibitor (PF-573228) were tested to identify an optimized dose that has a minimal effect on baseline myofibroblast activation but acts effectively in combination with Evogliptin (Figures 6B and 6C). We next sought to mimic the effects of IGFBP2 neutralization and assess if IGFBP2 promotes Evogliptin resistance through Rho/ROCK or FAK signaling. Using optimized doses, we demonstrated that the inhibition of Rho/ROCK or FAK signaling alone has a minimal impact on female VIC myofibroblast activation. However, combining these inhibitors of mechanosensing pathways with Evogliptin restores Evogliptin efficacy in female VICs (Figure 6D), mirroring the effects of IGFBP2 neutralization (Figures 4E-4H). Furthermore, male VICs cultured with male AVS serum were similarly responsive across all conditions tested (Figures 6E and 6F). Given the known ability of IGFBP2 to bind αvβ1 integrins,^58,59^ we assessed integrin subunit alpha V (ITGAV) and integrin subunit beta 1 (ITGB1) gene expression in VICs cultured on hydrogels, revealing increased *ITGAV* and *ITGB1* gene expression in female VICs relative to male VICs (Figure S12). Measuring the levels of secreted fibronectin yielded identical results, such as broad drug sensitivity in male VICs cultured with male serum and restored Evogliptin efficacy with the inhibition of Rho/ROCK or FAK signaling in female VICs cultured with female serum (Figures 6G and 6H). Additionally, culturing male and female VICs with serum from the opposite sex shows that IGFBP2-mediated Evogliptin resistance is primarily driven by serum composition rather than intrinsic cell sex differences (Figure S13). More specifically, female VICs cultured with male AVS serum mirrored male VIC responses, showing broad drug efficacy, while male VICs cultured with female AVS serum showed sensitivity to Evogliptin only when combined with Rho/ROCK or FAK inhibition.

## DISCUSSION

Collectively, our work revealed sex-specific associations between individual circulating serum factors and *in vitro* drug responses in male and female VICs. Although sex differences in individual serum factor abundances were minimal, the differences in correlations between serum factors and drug responses in male and female VICs were profound (Figure 3). This observation is consistent with prior work that identified sex-specific biomarkers that correlate with therapeutic outcomes in a multitude of other cardiovascular disease contexts.^60-62^ In our study, 19 robustly validated AVS biomarkers showed predominantly positive correlations with Ataciguat efficacy and predominantly negative correlations with Colchicine and Evogliptin efficacy in female VICs cultured with female AVS serum (Figure S4). In contrast, the opposite trends were observed in male VICs cultured with male AVS serum, highlighting fundamentally different biomarker and drug response relationships between sexes.

Notably, recent preliminary data from the Ataciguat clinical trial suggest that Ataciguat may be more effective at slowing calcification progression in male patients relative to female patients.^19^ In contrast, our *in vitro* data demonstrate greater efficacy of Ataciguat in reducing myofibroblast activation in female VICs cultured with female AVS serum (Figure 3). These differences underscore the need to better define the sex-specific mechanisms governing drug responses across experimental scales, which may arise from sex differences in differential protein binding, interactions with hormones, drug absorption, circulating metabolites, enzymatic activity, or drug pharmacokinetics.^63-66^ In contrast to Ataciguat, Colchicine and Evogliptin exhibited comparable efficacy in suppressing myofibroblast activation in male and female VICs. However, sex-specific responses to Colchicine and Evogliptin remain poorly characterized, and robust sex-stratified clinical trial data are lacking for both drugs. As sex is increasingly recognized as a key determinant of drug response, elucidating these underlying mechanisms may provide new opportunities for optimizing precision therapies for AVS.

Although baseline responses to Evogliptin differed minimally between sexes, our work identified a female-specific role for IGFBP2 as a predictive indicator for Evogliptin efficacy (Figure 3). Evogliptin works mechanistically by inhibiting dipeptidylpeptidase 4, which increases free IGF1 and IGF1 signaling to promote fibroblast quiescence.^67,68^ IGFBP2 and other IGFBPs counteract the inhibition of dipeptidylpeptidase 4 by binding to bioavailable IGF1 and inhibiting downstream IGF1 signaling.^69^ In female AVS serum, we observed an overall increased ratio of IGFBPs to IGF1 relative to male serum, suggesting lower baseline IGF1 bioavailability (Figure 4). Similar findings have been observed in healthy adults, where higher baseline ratios of IGFBPs to IGF1 were measured in female serum.^70^ This difference is particularly pronounced in adults over the age of 50, as menopause has been shown to further increase the ratio of IGFBPs to IGF1.^71^ Our analysis showed that altered serum IGFBP to IGF1 ratios are not driven by age in the AVS cohort, as is the case with the healthy cohort, suggesting that an imbalance of IGF1 signaling contributes to AVS incidence (Figure S9). Additionally, we suggest that this altered ratio of IGFBPs to IGF1 in female serum contributes to the observed female-specific IGFBP2-mediated resistance to Evogliptin.

**Figure 7.**
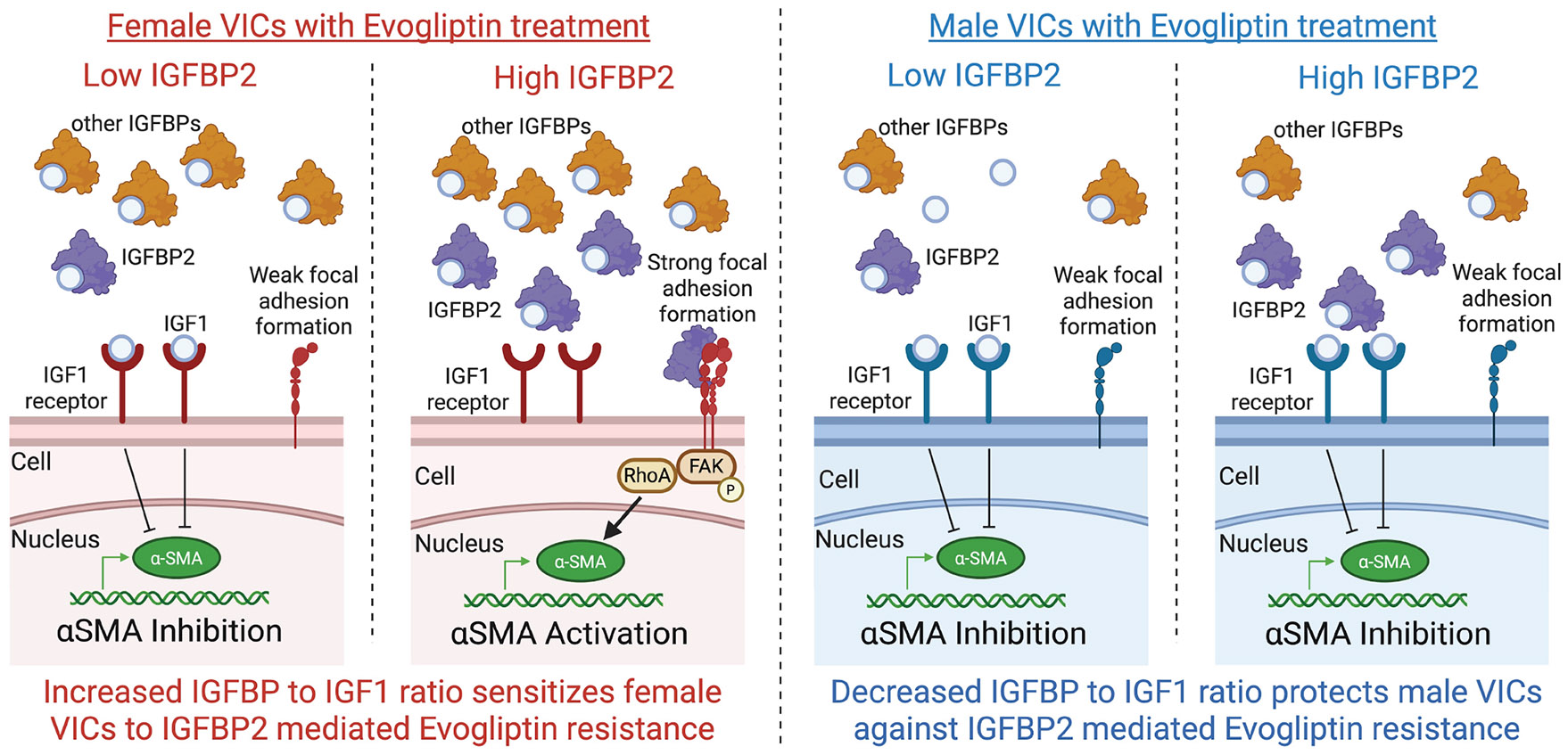
Proposed mechanism for IGFBP2 driving sex-specific Evogliptin resistance Summary mechanistic figure illustrates how elevated IGFBP2 in female serum modulates Evogliptin efficacy in female VICs. Figure created in BioRender. Aguado, B. (2026) https://BioRender.com/8zc6m9e.

Using our hydrogel cell culture platform, we further demonstrate that IGFBP2 acts through Rho/ROCK and FAK signaling to desensitize female VICs to Evogliptin treatment (Figure 6). Aside from directly binding IGF1, IGFBP2 can also alter cellular signaling through non-canonical pathways.^72^ Notably, IGFBP2 contains an RGD motif that allows it to directly bind αvβ1 integrins, which can help stabilize focal adhesion formation and promote downstream Rho/ROCK and FAK signaling.^58,59,72^ Our study showed that female VICs have higher baseline levels of *ITGAV* and *ITGB1* gene expression in response to culture on hydrogels, potentially providing more sites for IGFBP2 to bind (Figure S12). Aligning with these findings, our RNA sequencing analysis identified mechanosensing pathways such as integrin signaling and focal adhesion formation among the top pathways upregulated with IGFBP2 present in female AVS serum (Figure 5). Moreover, mechanosensing-linked pathways such as FAK signaling have been shown to directly modulate IGF1 signaling, highlighting potential reciprocal crosstalk between these pathways.^72,73^ In our data, several of the top differentially expressed genes (*ACTA1, ACTC1, MYH11,* and *MMP12*) are well-established downstream effectors of cytoskeletal tension and extracellular matrix remodeling that are functionally connected to IGF1 activity, supporting the existence of a feedback loop between mechanosensing and IGF1 signaling. Previous work has also shown that genes that escape X chromosome inactivation intersect with the Rho/ROCK signaling pathway, revealing a potential sex chromosome contribution to female-specific mechanosensing and subsequent Evogliptin resistance.^39^ Taken together, we propose that female serum has decreased bioavailable IGF1 and excess bioavailable IGFBP2, which enhances Rho/ROCK and FAK signaling to promote resistance to Evogliptin (Figure 7).

We also suggest that IGFBP2 may serve both as a biomarker of AVS progression and as a predictive indicator of drug responsiveness in female patients with AVS. IGFBP2 has emerged as a compelling AVS biomarker due to prior clinical studies that have demonstrated its ability to predict both AVS progression and patient outcomes following transcatheter aortic valve replacement.^56,74^ Specifically, patients with circulating IGFBP2 levels above 275 ng/mL were found to be at an elevated risk of mortality following aortic valve replacement. Although no sex stratification analysis was performed, this study highlights the clinical utility of IGFBP2 as a prognostic indicator for AVS risk stratification. Our work also builds upon reports implicating IGFBP2 in altered drug sensitivity and treatment outcomes in multiple disease contexts by extending the relevance of IGFBP2 to predicting antifibrotic drug outcomes in AVS.^75-77^ A recent large-scale proteomic analysis revealed a sex-specific association between IGFBP2 abundance and AVS progression, with elevated IGFBP2 correlating with disease severity only in female patients.^78^ These findings suggest a female-specific role for IGFBP2 in predicting treatment response in AVS and underscore the importance of considering biological sex when interpreting circulating biomarkers in AVS and other cardiovascular diseases.^79^

Together, we integrated serum proteomic profiling with hydrogel-based drug screening to establish predictive links between circulating biomarkers and antifibrotic drug responses in male and female VICs. We further identified IGFBP2 as a female-specific mediator of Evogliptin resistance that acts through Rho/ROCK and FAK signaling to counteract Evogliptin-induced suppression of myofibroblast activation. Our results also reveal that IGFBP2 may be a useful parameter for female AVS patient stratification for Evogliptin treatment in the future. More broadly, our results demonstrate that sex-stratified analysis of myofibroblast responses to circulating biomarkers can uncover mechanisms of drug resistance that would be missed in sex-aggregated studies, reinforcing the importance of incorporating sex as a biological variable in precision medicine strategies for AVS.

### Limitations of the study

Limitations to our study include the use of porcine VICs to identify a female VIC-specific association between serum IGFBP2 levels and Evogliptin efficacy, because of inherent challenges in obtaining low passage primary human VICs for culture. Fortunately, prior work has demonstrated that culturing porcine VICs with human AVS serum on hydrogels may serve as a bridge between *in vitro* disease modeling and *in vivo* patient outcomes.^39^ Future studies incorporating human-derived primary or induced pluripotent stem cell-derived VICs will be important to continue enhancing the clinical translatability of our findings.^80^ Additionally, even though our transcriptomic analysis, IGFBP2 antibody capture experiments, and small molecule inhibition studies provide a robust mechanistic framework across multiple biological replicates, we recommend future studies using gene knockdowns in VICs cultured on hydrogels to further probe Rho/ROCK and FAK signaling pathways as mediators of anti-fibrotic drug resistance. Beyond cell-specific mechanisms, the effects of organism-wide biological factors, including drug metabolism, hormone cycling, and complex physiological responses are also likely to contribute to sex-specific biomarker drug predictability.^81,82^ In addition, predicting drug responsiveness in AVS may be improved by integrating panels of multiple serum factors, such as an expanded profiling of global IGF1 signaling.^83,84^ Moreover, investigating the effects of therapeutics on VIC calcification using three-dimensional hydrogel culture systems can provide mechanistic insights into how inhibitors modulate osteoblast-like activation in male and female VICs.^45,85^ In sum, refinement of experimental models and biomarker frameworks may build upon our findings and provide additional context for sex-specific AVS patient treatment recommendations.

## STAR★METHODS

### EXPERIMENTAL MODEL AND STUDY PARTICIPANT DETAILS

#### Human serum collection and processing

Patient serum samples were obtained under the approval of the University of California San Diego Institutional Review Board (IRB #804209). AVS patients were seen in a multidisciplinary valve clinic and underwent evaluation by an interventional cardiologist using echocardiography. We collected 42 AVS patient serum samples (*N* = 25 male, *N* = 17 female) and 5 age-matched healthy serum samples (*N* = 3 male, *N* = 2 female). All patients signed an IRB-approved informed consent form, and all samples were de-identified.

Collected serum samples were allowed to sit at room temperature for 15-30 min to allow sufficient blood clotting. Serum samples were then centrifuged at 4°C and 2,000*g* for 10 min, placed on ice, and aliquoted for storage at −80°C for up to 3 years. Serum samples were discarded after two thaw cycles.

#### VIC isolation

All experiments using porcine heart samples were approved by the Environment, Health and Safety (EH&S) department at the University of California San Diego (Biohazard Use Authorization #2652). VICs were isolated from commercially available hearts sourced from 6 to 8-months-old male and female adult pigs (Midwest Research Swine).^86^ All female pigs have not previously given birth. Biological replicates are defined as a pooled sample of VICs isolated from a minimum of three sex-matched porcine hearts. To summarize, aortic valve leaflets were isolated, pooled by sex, and submerged into Earle’s Balanced Salt Solution (EBSS, Sigma-Aldrich) supplemented with 1 μg/mL amphotericin B (Thermo Fisher), 50 U/mL penicillin and 50 μg/mL streptomycin (Sigma, Cat. No. P4458). After all leaflets were isolated, fresh EBSS supplemented with 250 units/mL of type II collagenase (Worthington) was added and leaflets were shaken for 30 min at 37°C and 5% carbon dioxide. Next, leaflets were vortexed for 30 s then placed in fresh EBSS/collagenase solution. Following a 60-min incubation at 37°C and 5% carbon dioxide while shaking, leaflets were vortexed for two minutes and pressed through a 100 μm cell strainer. VICs were pelleted through a 10-min centrifugation at 200*g*, then resuspended in VIC growth media (Media 199 (Life Technologies), 15% fetal bovine serum (FBS; Life Technologies, Cat. No. 16000069), 1 μg/mL amphotericin B, 50 U/mL penicillin, and 50 μg/mL streptomycin). Lastly, VICs were seeded on T75 Falcon tissue culture treated flasks (Fisher) at 37°C and 5% carbon dioxide for expansion. Fresh VIC culture media was added every 24 to 48 h until VICs were ~80% confluent. For long term storage, VICs were frozen down overnight at −80°C in 1 mL aliquots of 50% FBS, 45% VIC growth media, and 5% DMSO then transferred to a cell dewar for long-term storage in liquid nitrogen. Similarity between VIC isolations were confirmed by quantifying baseline αSMA expression in response to two-day culture on hydrogels. Multiple aliquots of a single FBS batch were aliquoted and frozen at −20°C for long term storage. As needed, fresh aliquots of the same FBS batch were thawed and used to prepare additional media.

### METHOD DETAILS

#### SomaScan proteomic analysis

All 47 serum samples were submitted for proteomic analysis using the SomaScan DNA aptamer array following protocols outlined previously. An additional 38 age-matched healthy serum samples (*N* = 16 male, *N* = 22 female) were used from the public SomaScan repository to supplement our analyses. A serum comparison analysis pipeline was developed in R (version 2023.06.1) to quantify patient differences in serum factor abundance. Briefly, the raw SomaScan data was trimmed and filtered to remove strong outliers (defined as quartile 1–3 * interquartile range and quartile 3 + 3 * interquartile range). A principal component analysis and unsupervised hierarchical clustering analysis were then performed to assess global differences in patient serum proteomes. Serum samples were then grouped by variables of interest (i.e., healthy vs. diseased or male vs. female) and compared via unpaired *t* test to generate false discovery rate (FDR) adjusted *p*-values and fold changes for every serum factor (*N* = 1,512) using the standard t.test function and the Benjamini-Hochberg method for FDR adjusted *p*-value correction. Significant proteins were defined as FDR-adjusted *p*-value less than 0.05 and a fold change greater than 1.2.

#### Hydrogel formation

Hydrogels were generated in high throughput as described previously.^47^ Briefly, 96-well glass bottom well plates (Cellvis, Cat. No. P96-1.5H-N) were placed in a lightly sealed autoclave jar with an open scintillation vial containing 100 μL of mercaptopropyl-trimethoxysilane (Sigma-Aldrich, Cat. No. 175617) and heated in an oven at 60°C for a minimum of 6 h. Next, hydrogel precursor solution was prepared using phosphate buffered saline (PBS, Invitrogen, Cat. No. 14190-250), 7% wt/vol 8-arm 20 kDa polyethylene glycolnorbornene (PEG-Nb),^57^ 5 kDa PEG-dithiol (Jenkem, Cat. No. A4075-5), CRGDS cell binding motif (Bachem), and lithium phenyl-2,4,6-trimethyl-benzoylphosphinate (Fisher Scientific, Cat. No. 900889). 7 μL of hydrogel precursor solution was added directly into the center of each well in the 96-well glass bottom well plate using an EPMotion 5073L automated liquid handling system (Eppendorf). Using a custom 3D-printed stamp with glass rods, the hydrogel precursor solution was stamped flat at a height of 250 μm, inverted, and cured under 10 mW/cm^2^ of ultraviolet (UV) light for 3 min. The gel stamp was then removed, and newly formed hydrogels were washed with 5% isopropyl alcohol in PBS for 20 min. Next, hydrogels were washed twice with PBS then incubated in VIC culture media (Media199 (Life Technologies, Cat. No. 11-043-023), 1% FBS or 1% AVS patient serum, 1 μg mL amphotericin B (Thermo Fisher, Cat. No. 15290026), 50 U/mL penicillin and 50 μg/mL streptomycin (Sigma, Cat. No. P4458)) overnight at 37°C for experimental use the following day.

For RNA sequencing and RT-qPCR experiments, 65 μL of gel precursor solution was pipetted onto a Sigmacote (Sigma, Cat. No. SL2) treated glass slide, then covered with a thiolated 25-mm circular glass coverslip and cured under 10 mW/cm^2^ of ultraviolet (UV) light for 3 min. Gels were then removed from the glass slide, placed in an untreated 6-well plate (Fisher, Cat. No. 08-772-49) and sterilized following the same wash protocol outlined above.

#### VIC culture

Frozen aliquots of passage 1 male or female VICs were thawed and immediately resuspended in VIC growth media. Cells were pelleted through a 5-min centrifugation at 200*g* and resuspended in 4 mL of fresh VIC growth media. The cell suspension was then split and seeded into two wells in a Falcon tissue culture treated 6 well plate (Fisher, Cat. No. 08-772-49) for expansion. Media changes were performed every 24–48 h until VICs achieved 80% confluency. Cells were then collected by removing the spent media, rinsing with PBS for 1 min, then incubating each well with 1 mL of 1× trypsin (Gibco, Cat. No. 15400054) for 3 min at 37°C and 5% carbon dioxide. VIC growth media was added in equal volume to neutralize the trypsin in each well and the two wells were pooled together. Cells were again pelleted through a 5-min centrifugation at 200*g* then resuspended in 1 mL of VIC culture media. To ensure even cell confluency for experiments, VICs were counted using an automated hemocytometer then seeded at 6400 cells/well (20,000 cells/cm^2^) for immunostaining experiments and 180,000 cells/well for RT-qPCR and RNA sequencing experiments.

#### Immunostaining

First, VICs were fixed in a solution of 4% paraformaldehyde solution in PBS (80 μL per well) for 20 min. Next, VICs were permeabilized using a solution of 0.1% Triton X-100 (Fisher, Cat. No. AC215682500) in PBS (150 μL per well) for 1 h and blocked with 5% bovine serum albumin in PBS (150 μL per well) for 1 h. Depending on experimental setup, immunostaining was performed using a mouse anti-αSMA primary antibody (Abcam, Cat. No. AB7817, 1:300, RRID: AB_262054) and/or a rabbit anti-cleaved caspase 3 primary antibody (Cell Signaling Technology, Cat. No. 9661S, 1:250, RRID: AB_2341188) in blocking solution (80 μL per well) for one hour at room temperature. Primary antibody solution was stored at −20°C and reused up to four times. After a 10 min wash in PBS with 0.05% Tween 20 (150 μL per well) (Sigma, Cat No. P1379), secondary staining was performed for 1 h in the dark using a solution of PBS with goat anti-mouse Alexa Fluor 488 (80 μL per well) (Life Technologies, Cat No. A11001, 1:300 dilution, RRID: AB_2534069), goat anti-rabbit Alexa Fluor 647 (Life Technologies, Cat No. A21245, 1:300 dilution, RRID: AB_2535813), 4′-6-diamidino-2-phenylindole (Sigma, Cat No. 10236276001, 1:500 dilution) and HCS Cell Mask Deep Orange (Life Technologies, Cat. No. H32713, 1:5000 dilution). Lastly, VICs were washed once with PBS (150 μL per well) for 10 min then stored at 4°C in fresh PBS (150 μL per well) in the dark until imaging. Cells were imaged within 1 week of immunostaining using a Nikon Eclipse Ti2-E with uniform automated imaging settings.

#### Calculating αSMA reduction and cytotoxicity

A custom MATLAB script was used to quantify single cell αSMA gradient mean intensity data from immunostained cells. Drug efficacy was then calculated as the percent reduction in single cell αSMA gradient mean intensity of the treated condition normalized to a vehicle control as shown in the equation below:

$$\%\;\alpha \text{SMA reduction}=100\times \left(\frac{Control-Drug\;Condition}{Control}\right)$$

Cytotoxicity was assessed by assigning each cell in an image as alive or dead based on immunofluorescent cleaved caspase-3 expression normalized to cell mask. A positive control for cell death (10 nM Staurosporine (Cell Signaling Technologies, Cat. No. 9953S) in VIC culture media) was used to set the cleaved caspase-3 threshold for a live/dead cell. For all αSMA and cytotoxicity data, a minimum of 100 cells were used per well with multiple wells per condition.

#### Inhibitor and serum factor correlation analysis

Using R, we developed an analysis pipeline to correlate *in vitro* drug efficacy with individual serum factor abundance. Serum factor RFU values were log10 transformed and filtered to remove strong outliers as described earlier. Patient serum data was then sex-separated to perform a sex-specific correlation analysis or pooled to perform a global correlation analysis. For each analysis, the percent αSMA reduction for Ataciguat, Evogliptin, and Colchicine were aligned with individual serum factor relative fluorescent unit (RFU) values for each patient. The drug efficacy and corresponding serum factor RFU values were then plotted for each serum sample to generate a total of 31 distinct data points (16 male and 15 female). Using the corbetw2mat function in R (lineup package, version 0.44), the correlation coefficient and corresponding *p*-value for each drug-serum factor combination were calculated. Significant correlations were calculated for sex-separated and combined analysis and were defined as *p*-value less than 0.05.

#### RT-qPCR

25 mm hydrogels seeded with VICs were inverted into cell lysis buffer (Qiagen RNEasy Micro Kit, Cat. No. 74004) for two minutes. The hydrogels were then flipped over and rinsed with 70% ethanol. The solution was then collected in spin columns. RNA was collected for all samples following manufacturers guidelines and tested for quality using a NanoDrop 2000 spectrophotometer (Thermo Fisher). RNA was then converted to cDNA using an iScript Synthesis kit (Bio-Rad). Relative RNA expression was quantified using IQ SYBR Green Supermix (Bio-Rad) fluorescent intensity measured on a CFX384 iCycler (Bio-Rad). Genes were normalized to the *RPL30* gene and quantified using the ΔΔCT method. Forward and reverse primer sequences can be found in Table S6.

##### Protein and inhibitor preparation

Lyophilized proteins were stored at −20°C for up to 6 months and resuspended following manufacturer’s guidelines. Recombinant human insulin-like growth factor-binding protein 2 (IGFBP2, R&D systems, Cat. No. 674-B2-025) was aliquoted at 100 μg/mL in sterile PBS. IGFBP2 stocks were stored at −20°C for up to 3 months and discarded after two thaw cycles.

Lyophilized inhibitors were stored at −20°C for up to 1 month then resuspended following manufacturer’s guidelines. Evogliptin (MedChem Express, Cat. No. HY-117985B), Ataciguat (MedChem Express, Cat. No. HY-17500), and PF-573228 (MedChem Express, Cat. No. HY-10461) were resuspended in sterile dimethyl sulfoxide (DMSO) (Fisher, Cat. No. D2438). Colchicine (MedChem Express, Cat. No. HY-16569) and H-1152 (Bio-techne, Cat. No. 2414) were resuspended in sterile deionized water. All aliquots were stored at −80°C for up to 6 months and discarded after two thaw cycles. Evogliptin, Ataciguat, and PF-573228 were diluted prior to experimental use to ensure a final DMSO concentration below 0.3%.

#### Enzyme-linked immunosorbent assays (ELISAs)

Serum concentrations of IGFBP2 were quantified using an IGFBP2 enzyme-linked immunosorbent assay (ELISA) kit (Invitrogen, Cat. No. EHIGFBP2). Following manufacturer’s guidelines, serum samples were diluted 1:500 in assay diluent A and standards were prepped using lyophilized IGFBP2 resuspended to concentrations ranging from 6000 pg/mL to 8.23 pg/mL following a 3-fold dilution. 100 μL of diluted serum samples, standard curves, and controls were added to wells containing an IGFBP2 capture antibody overnight at 4°C while shaking. Wells were then washed three times with wash buffer (300 μL) then incubated with 100 μL of biotin conjugate for 1 h while shaking. The wash step was then repeated before adding 100 μL of streptavidin-HRP solution (Invitrogen) for 45 min with gentle shaking. The wash step was repeated one last time, then 100 μL of TMB substrate (Invitrogen) was added for 30 min in the dark while shaking. Finally, 50 μL of stop solution (Invitrogen) was added and the plate was immediately imaged at 450 nm using a PerkinElmer Envision plate reader. The data from the standards was fed into GraphPad Prism (Version 11.0.0) to generate a standard curve. The standard curve was then used to convert OD450 values to IGFBP2 concentrations in each serum sample, followed by multiplication with the serum dilution factor (500).

Fibronectin secretion was quantified using an ELISA kit (R&D systems, Cat. No. DY1918-05) as an additional assay to assess myofibroblast activation.^87^ After two days of treatment, cell culture supernatant was collected and analyzed following manufacturer’s guidelines. Supernatant was diluted 1:100 in reagent diluent and standards were prepped using lyophilized Fibronectin resuspended to concentrations ranging from 200 ng/mL to 3.125 ng/mL following a 2-fold serial dilution. After performing the ELISA, absorbance readings were taken at 450 nm and background signal was removed by subtracting blank controls and readings taken at 540 nm. To confirm that all quantified Fibronectin was of cellular origin, acellular supernatant was measured and subtracted from cell culture supernatant readings. All quantified Fibronectin secretion values were normalized by the number of cells seeded per well.

#### Neutralizing antibody experiments

Human IGFBP2 neutralizing antibody (R&D systems, Cat. No AF674, RRID: AB_355517) and normal IgG control antibody (R&D systems, Cat. No AB-108-C, RRID: AB_354267) were resuspended in sterile PBS at 200 μg/mL and 1,000 μg/mL respectively. Antibodies were stored at −20°C for up to 6 months and discarded after two thaw cycles. IGFBP2 neutralizing antibody and normal IgG control antibody were added at 1 μg/mL to media containing AVS patient serum and incubated at 37°C for 1 h to allow for neutralization prior to cell seeding.

#### RNA sequencing

RNA was isolated for sequencing following the protocol described above and assessed for quality using an Agilent TapeStation 4200. A minimum of 100 ng of RNA per sample was submitted to The Sanford Consortium for Regenerative Medicine Genomics Core, which generated cDNA libraries and performed indexing. RNA sequences were assessed for quality using fastqc, and adapter sequences were removed using trimomatic (v0.39) if needed. Samples were then aligned to the porcine genome (SusScrofa11.1) using HISAT2 (v2.2.1) and trimmed to remove genes with counts less than 10. The original sequencing files can be accessed through the National Center for Biotechnology Information (NCBI) Gene Expression Omnibus (GEO) repository using accession number GSE316678.

Differential gene expression analysis was performed in R. Briefly, RNA samples were grouped by experimental condition and compared via unpaired *t* test to generate false discovery rate (FDR) adjusted *p*-values and fold changes for every gene measured. Significant genes were calculated using the standard t.test function and the Benjamini-Hochberg method for FDR adjusted *p*-value correction and defined as FDR-adjusted *p*-value less than 0.05 and a fold change greater than 2. Principal component analysis and pathway enrichment were performed as described previously.

#### Pathway enrichment analysis

A spreadsheet containing Entrez gene IDs, fold changes between groups, and FDR-adjusted *p*-values was uploaded into the Ingenuity pathway analysis (IPA) software (Qiagen). A core expression analysis was performed using differentially expressed proteins (FDR <0.05 and fold change greater than 1.2) or genes (FDR <0.05 and fold change greater than 2) and the resultant signaling pathways were analyzed. Significance was determined using the standard t.test function and the Benjamini-Hochberg method for FDR adjusted *p*-value correction. This analysis was repeated to characterize differences between healthy and AVS patient proteomes and female VICs treated with Evogliptin with and without an IGFBP2 neutralizing antibody. Kyoto Encyclopedia of Genes and Genomes (KEGG) pathway enrichment analysis was also performed on differentially abundant proteins and genes using the ClusterProfiler package (version 4.12.6) in R.

### QUANTIFICATION AND STATISTICAL ANALYSIS

Unless otherwise stated, statistical analysis was performed in GraphPad (Version 11.0.0) and significance was defined as a *p*-value less than 0.05. Comparisons between two groups were calculated by an unpaired *t* test with Welch’s correction and comparisons between multiple groups were calculated via one-way, two-way, or three-way ANOVA with Tukey posttests. Unless stated, all data are shown as mean ± standard deviation.

## Supplementary Material

1

2

3

Supplemental information can be found online at https://doi.org/10.1016/j.celrep.2026.117678.

## Figures and Tables

**Table:** KEY RESOURCES TABLE

REAGENT or RESOURCE	SOURCE	IDENTIFIER
Antibodies		
Mouse monoclonal anti-αSMA	Abcam	Cat# AB7817; RRID: AB_262054
Rabbit monoclonal anti-cleaved caspase 3	Cell Signaling Technology	Cat#9661S; RRID: AB_2341188
Goat anti-mouse Alexa Fluor 488	Life Technologies	Cat#A11001; RRID: AB_2534069
Goat anti-rabbit Alexa Fluor 647	Life Technologies	Cat# A21245; RRID: AB_2535813
IGFBP2 neutralizing antibody	R&D systems	Cat# AF674; RRID: AB_355517
Normal IgG control antibody	R&D systems	Cat# AB-108-C; RRID: AB_354267
Biological samples		
Patient serum samples	Sulpizio Cardiovascular Center, UCSD	IRB #804209
Chemicals, peptides, and recombinant proteins		
Mercaptopropyltrimethoxysilane	Sigma-Aldrich	175617
Lithium phenyl-2,4,6-trimethyl-benzoylphosphinate	Fisher Scientific	900889
Sigmacote	Sigma-Aldrich	SL2
IGFBP2	R&D systems	674-B2-025
Evogliptin	MedChem Express	HY-117985B
Ataciguat	MedChem Express	HY-17500
PF-573228	MedChem Express	HY-10461
Colchicine	MedChem Express	HY-16569
H-1152	Bio-techne	2414
Critical commercial assays		
IGFBP2 ELISA kit	Invitrogen	EHIGFBP2
Fibronectin ELISA kit	R&D systems	DY1918-05
Deposited data		
SomaScan serum repository	SomaLogic	https://menu.somalogic.com/file-downloads/population-sets
Biomarkers for Aortic Valve Stenosis	Shelbaya et al.^56^	https://doi.org/10.1016/j.jacc.2023.11.021
RNA sequencing samples	This paper	NCBI GEO Accession Number: GSE316678 https://www.ncbi.nlm.nih.gov/geo/query/acc.cgi?acc=GSE316678
Experimental models: Cell lines		
Male porcine VICs	This paper	N/A
Female porcine VICs	This paper	N/A
Oligonucleotides		
Primer: RPL30 Forward: AGATTTCCTCAAGGCTGGGC	This paper	N/A
Primer: RPL30 Reverse: GCTGGGGTACAAGCAGACTC	This paper	N/A
Primer: ACTA2 Forward: GCAAACAGGAATACGATGAAGCC	This paper	N/A
Primer: ACTA2 Reverse: AACACATAGGTAACGAGTCAGAGC	This paper	N/A
Primer: ITGAV Forward: ATGCAACAGGCAACAGAGACT	This paper	N/A
Primer: ITGAV Reverse: TCCTGAAGAAAGCAAGTTCCA	This paper	N/A
Primer: ITGB1 Forward: CAAGCAGGGCCAAATTGTGG	This paper	N/A
Primer: ITGB1 Reverse: CCTTTGCTACGGTTGGTTACATT	This paper	N/A
Software and algorithms		
R	R Foundation	https://www.r-project.org
Ingenuity Pathway Analysis	Qiagen	https://analysis.ingenuity.com/pa/installer/select
MATLAB	MathWorks	https://www.mathworks.com/products/matlab.html
Biorender	Biorender	Biorender.com
Prism 11	GraphPad	https://www.graphpad.com/

## Highlights

- Male and female patients with aortic valve stenosis have distinct serum biomarkers
- Female serum IGFBP2 predicts Evogliptin efficacy in valvular interstitial cells
- IGFBP2 counteracts Evogliptin through mechanosensing pathways
- IGFBP2 may serve as a female-specific biomarker to predict Evogliptin response

## References

[1] Aikawa E, and Hutcheson JD (2022). The Developmental Origin of Calcific Aortic Stenosis. N. Engl. J. Med. 386, 1372–1374. 10.1056/nejmcibr2200439. 35388675

[2] Miller JD, Weiss RM, and Heistad DD (2011). Calcific Aortic Valve Stenosis: Methods, Models, and Mechanisms. Circ. Res. 108, 1392–1412. 10.1161/CIRCRESAHA.110.234138. 21617136 PMC3150727

[3] Călin A, Roşca M, Beladan CC, Enache R, Mateescu AD, Ginghină C, and Popescu BA (2015). The left ventricle in aortic stenosis – imaging assessment and clinical implications. Cardiovasc. Ultrasound 13, 22. 10.1186/s12947-015-0017-4. 25928763 PMC4425891

[4] Osnabrugge RLJ, Mylotte D, Head SJ, Van Mieghem NM, Nkomo VT, LeReun CM, Bogers AJJC, Piazza N, and Kappetein AP (2013). Aortic Stenosis in the Elderly Disease Prevalence and Number of Candidates for Transcatheter Aortic Valve Replacement: A Meta-Analysis and Modeling Study. J. Am. Coll. Cardiol. 62, 1002–1012. 10.1016/j.jacc.2013.05.015. 23727214

[5] Danielsen R, Aspelund T, Harris TB, and Gudnason V (2014). The prevalence of aortic stenosis in the elderly in Iceland and predictions for the coming decades: The AGES-Reykjavik study. Int. J. Cardiol. 176, 916–922. 10.1016/j.ijcard.2014.08.053. 25171970 PMC4742571

[6] Wang B, Mei Z, Yang H, Gao W, Ma L, and An G (2025). Global, regional, and national burden of nonrheumatic calcific aortic valve disease based on GBD study 2021. Sci. Rep. 15, 29464. 10.1038/s41598-025-14522-x. 40790322 PMC12340002

[7] Fact.MR (2024). Aortic Stenosis Treatment Market Size, Share, Growth, and Forecast (2024-2034) (Fact.MR). https://www.factmr.com/report/aortic-stenosis-treatment-market.

[8] Daubert MA, Weissman NJ, Hahn RT, Pibarot P, Parvataneni R, Mack MJ, Svensson LG, Gopal D, Kapadia S, Siegel RJ, (2017). Long-Term Valve Performance of TAVR and SAVR. JACC, Cardiovasc. Imaging 10, 15–25. 10.1016/j.jcmg.2016.11.004. 28017714

[9] Gozdek M, Raffa GM, Suwalski P, Kołodziejczak M, Anisimowicz L, Kubica J, Navarese EP, and Kowalewski M; SIRIO-TAVI group (2018). Comparative performance of transcatheter aortic valve-in-valve implantation versus conventional surgical redo aortic valve replacement in patients with degenerated aortic valve bioprostheses: systematic review and meta-analysis. Eur. J. Cardio. Thorac. Surg. 53, 495–504. 10.1093/ejcts/ezx347. 29029105

[10] Kumar A, Sato K, Narayanswami J, Banerjee K, Andress K, Lokhande C, Mohananey D, Anumandla AK, Khan AR, Sawant AC, (2018). Current Society of Thoracic Surgeons Model Reclassifies Mortality Risk in Patients Undergoing Transcatheter Aortic Valve Replacement. Circulation. Circ. Cardiovasc. Interv. 11, e006664. 10.1161/circinterventions.118.006664. 30354591

[11] Goleski PJ, Reisman M, and Don CW (2018). Reversible thrombotic aortic valve restenosis after valve-in-valve transcatheter aortic valve replacement. Cathet. Cardiovasc. Interv. 91, 165–168. 10.1002/ccd.26522. 27198960

[12] Aksoy O, Cam A, Goel SS, Houghtaling PL, Williams S, Ruiz-Rodriguez E, Menon V, Kapadia SR, Tuzcu EM, Blackstone EH, and Griffin BP (2012). Do Bisphosphonates Slow the Progression of Aortic Stenosis? J. Am. Coll. Cardiol. 59, 1452–1459. 10.1016/j.jacc.2012.01.024. 22497825

[13] Pawade TA, Doris MK, Bing R, White AC, Forsyth L, Evans E, Graham C, Williams MC, van Beek EJR, Fletcher A, (2021). Effect of Denosumab or Alendronic Acid on the Progression of Aortic Stenosis: A Double-Blind Randomized Controlled Trial. Circulation 143, 2418–2427. 10.1161/CIRCULATIONAHA.121.053708. 33913339 PMC8212878

[14] Chan KL, Teo K, Dumesnil JG, Ni A, and Tam J (2010). Effect of Lipid Lowering With Rosuvastatin on Progression of Aortic Stenosis. Circulation 121, 306–314. 10.1161/CIRCULATIONAHA.109.900027. 20048204

[15] Cowell SJ, Newby DE, Prescott RJ, Bloomfield P, Reid J, Northridge DB, and Boon NA; Scottish Aortic Stenosis and Lipid Lowering Trial, Impact on Regression SALTIRE Investigators (2005). A Randomized Trial of Intensive Lipid-Lowering Therapy in Calcific Aortic Stenosis. N. Engl. J. Med. 352, 2389–2397. 10.1056/NEJMoa043876. 15944423

[16] Rossebø AB, Pedersen TR, Boman K, Brudi P, Chambers JB, Egstrup K, Gerdts E, Gohlke-Bärwolf C, Holme I, Kesäniemi YA, (2008). Intensive Lipid Lowering with Simvastatin and Ezetimibe in Aortic Stenosis. N. Engl. J. Med. 359, 1343–1356. 10.1056/NEJMoa0804602. 18765433

[17] Scandinavian Simvastatin Survival Study Group (1994). Randomised trial of cholesterol lowering in 4444 patients with coronary heart disease: the Scandinavian Simvastatin Survival Study (4S). Lancet 344, 1383–1389. 10.1016/S0140-6736(94)90566-5. 7968073

[18] Rajamannan NM, Evans FJ, Aikawa E, Grande-Allen KJ, Demer LL, Heistad DD, Simmons CA, Masters KS, Mathieu P, O’Brien KD, (2011). Calcific Aortic Valve Disease: Not Simply a Degenerative Process. Circulation 124, 1783–1791. 10.1161/CIRCULATIONAHA.110.006767. 22007101 PMC3306614

[19] Zhang B, Enriquez-Sarano M, Schaff HV, Michelena HI, Roos CM, Hagler MA, Zhang H, Casaclang-Verzosa G, Huang R, Bartoo A, (2025). Reactivation of Oxidized Soluble Guanylate Cyclase as a Novel Treatment Strategy to Slow Progression of Calcific Aortic Valve Stenosis: Preclinical and Randomized Clinical Trials to Assess Safety and Efficacy. Circulation 151, 913–930. 10.1161/CIRCULATIONAHA.123.066523. 39989354

[20] Song J-K, Lee S, Kim Y-J, Kim H-K, Ha J-W, Choi E-Y, Park S-W, Park S-J, Park Y-H, Park J-H, (2024). Effect of Evogliptin on the Progression of Aortic Valvular Calcification. J. Am. Coll. Cardiol. 84, 1064–1075. 10.1016/j.jacc.2024.06.037. 39260927

[21] Mohammadnia N, Vestjens LTW, Craig NJ, Tijssen JGP, Knol RJJ, Lazarenko SV, Duffels MGJ, Jaspers Focks J, Hemels MEW, Oving I, (2025). The effects of low-dose colchicine on the progression of aortic valve stenosis: Rationale, design, and baseline characteristics of the Colchicine and Inflammation in Aortic Stenosis (CHIANTI) trial. Am. Heart J. 290, 297–309. 10.1016/j.ahj.2025.07.010. 40659197

[22] Hopfgarten J, James S, Lindhagen L, Baron T, Ståhle E, and Christersson C (2024). Medical treatment of heart failure with renin–angiotensin–aldosterone system inhibitors and beta-blockers in aortic stenosis: association with long-term outcome after aortic valve replacement. Eur. Heart J. Open 4, oeae039. 10.1093/ehjopen/oeae039. 38812477 PMC11135942

[23] Small AM, Yang T-Y, Itoh S, Thériault S, Dufresne L, Kurosawa R, Komuro I, Matsuda K, Vy HMT, Farber-Eger EH, (2026). Genomic and transcriptomic analyses of aortic stenosis enhance therapeutic target discovery and disease prediction. Nat. Genet. 58, 57–66. 10.1038/s41588-025-02417-6. 41419686 PMC12807872

[24] Félix Vélez NE, Gorashi RM, and Aguado BA (2022). Chemical and molecular tools to probe biological sex differences at multiple length scales. J. Mater. Chem. B 10, 7089–7098. 10.1039/D2TB00871H. 36043366 PMC9632480

[25] Aguado BA, Grim JC, Rosales AM, Watson-Capps JJ, and Anseth KS (2018). Engineering precision biomaterials for personalized medicine. Sci. Transl. Med. 10, eaam8645. 10.1126/scitranslmed.aam8645. 29343626 PMC6079507

[26] Fogg K, Tseng NH, Peyton SR, Holeman P, Mc Loughlin S, Fisher JP, Sutton A, Shikanov A, Gnecco JS, Knight KM, (2022). Roadmap on biomaterials for women’s health. J. Phys. Mater. 6, 012501. 10.1088/2515-7639/ac90ee. PMC13218746 42220979

[27] Simard L, Côté N, Dagenais F, Mathieu P, Couture C, Trahan S, Bossé Y, Mohammadi S, Pagé S, Joubert P, and Clavel MA (2017). Sex-Related Discordance Between Aortic Valve Calcification and Hemodynamic Severity of Aortic Stenosis. Circ. Res. 120, 681–691. 10.1161/CIRCRESAHA.116.309306. 27879282

[28] Voisine M, Hervault M, Shen M, Boilard AJ, Filion B, Rosa M, Bossé Y, Mathieu P, Côté N, and Clavel MA (2020). Age, Sex, and Valve Phenotype Differences in Fibro-Calcific Remodeling of Calcified Aortic Valve. J. Am. Heart Assoc. 9, e015610. 10.1161/JAHA.119.015610. 32384012 PMC7660864

[29] Porras AM, McCoy CM, and Masters KS (2017). Calcific Aortic Valve Disease. Circ. Res. 120, 604–606. 10.1161/circresaha.117.310440. 28209788 PMC5319688

[30] Parikh PB, Wang TY, Sharma N, Kort S, Skopicki HA, Gruberg L, Jeremias A, Pyo R, Chikwe J, Butler J, (2020). Sex-Related Differences in Early- and Long-Term Mortality After Transcatheter and Surgical Aortic Valve Replacement: A Systematic Review and Meta-Analysis. J. Invasive Cardiol. 32, 295–301. 32198317 10.25270/jic/19.00371

[31] He JJ, Shah M, Vemulapalli S, Kosinski AS, Mack MJ, Leon MB, Liao XX, and Zhuang XD (2025). Association between sex and transcatheter aortic valve replacement outcome for bicuspid and tricuspid aortic stenosis. Eur. Heart J. 46, ehaf784.2367. 10.1093/eurheartj/ehaf784.2367.

[32] Guthoff H, Abdel-Waha M, Kim W-K, Wienemann H, Shamekhi J, Eckel C, Von Der Heide I, Veulemans V, Landt M, Schewel J, (2026). Sex differences in hemodynamics and outcomes after transcatheter aortic valve replacement. Clin. Res. Cardiol. 115, 1207–1217. 10.1007/s00392-025-02794-2. 41264012 PMC13249733

[33] Crousillat D, Shariff D, Duan R, Tanguturi V, and Elmariah S (2025). Sex Differences in the Clinical Recognition of Significant Aortic Stenosis. JACC. Adv. 4, 102370. 10.1016/j.jacadv.2025.102370. 41447283 PMC12686702

[34] Thevathasan T, Reitz F, Hornig C, Leistner DM, Dreger H, Sündermann S, Mattig I, Unbehaun A, Haghikia A, Skurk C, (2025). Sex Differences in Clinical and Patient-Reported Outcomes in Transcatheter Aortic Valve Implantation. JACC. Adv. 4, 102012. 10.1016/j.jacadv.2025.102012. 40682895 PMC12301741

[35] Goliasch G, and Lang IM (2021). Impact of sex on the management and outcome of aortic stenosis patients: a female aortic valve stenosis paradox, and a call for personalized treatments? Eur. Heart J. 42, 2692–2694. 10.1093/eurheartj/ehab331. 34128046 PMC8282323

[36] Raddatz MA, Gonzales HM, Farber-Eger E, Wells QS, Lindman BR, and Merryman WD (2020). Characterisation of aortic stenosis severity: a retrospective analysis of echocardiography reports in a clinical laboratory. Open Heart 7, e001331. 10.1136/openhrt-2020-001331. 32817269 PMC7437881

[37] Toyofuku M, Taniguchi T, Morimoto T, Yamaji K, Furukawa Y, Takahashi K, Tamura T, Shiomi H, Ando K, Kanamori N, (2017). Sex Differences in Severe Aortic Stenosis – Clinical Presentation and Mortality. Circ. J. 81, 1213–1221. 10.1253/circj.cj-16-1244. 28392546

[38] Kjeldsen EW, Thomassen JQ, Rasmussen KL, Nordestgaard BG, Tybjærg-Hansen A, and Frikke-Schmidt R (2025). Cardiovascular risk factors and aortic valve stenosis: towards 10-year absolute risk charts for primary prevention. Eur. J. Prev. Cardiol. 32, 1684–1693. 10.1093/eurjpc/zwae177. 38775783

[39] Aguado BA, Walker CJ, Grim JC, Schroeder ME, Batan D, Vogt BJ, Rodriguez AG, Schwisow JA, Moulton KS, Weiss RM, (2022). Genes That Escape X Chromosome Inactivation Modulate Sex Differences in Valve Myofibroblasts. Circulation 145, 513–530. 10.1161/circulationaha.121.054108. 35000411 PMC8844107

[40] Baddour T, Ninh VK, Gorashi RM, Peña R, King KR, and Aguado BA (2026). Spatial and Single-Cell Mapping Reveals Valvular Interstitial Cell and Macrophage Sex Differences in Calcific Aortic Valve Disease. ATVB, ATVBAHA 126, 324694. 10.1161/ATVBAHA.126.324694. PMC13220916 42206364

[41] Mabry KM, Lawrence RL, and Anseth KS (2015). Dynamic stiffening of poly(ethylene glycol)-based hydrogels to direct valvular interstitial cell phenotype in a three-dimensional environment. Biomaterials 49, 47–56. 10.1016/j.biomaterials.2015.01.047. 25725554 PMC4346780

[42] Félix Vélez NE, Tu K, Guo P, Reeves RR, and Aguado BA (2025). Secreted Cytokines From Inflammatory Macrophages Modulate Sex Differences in Valvular Interstitial Cells on Hydrogel Biomaterials. J. Biomed. Mater. Res. 113, e37885. 10.1002/jbm.a.37885. PMC11875511 39995146

[43] Hjortnaes J, Camci-Unal G, Hutcheson JD, Jung SM, Schoen FJ, Kluin J, Aikawa E, and Khademhosseini A (2015). Directing Valvular Interstitial Cell Myofibroblast-Like Differentiation in a Hybrid Hydrogel Platform. Adv. Healthcare Mater. 4, 121–130. 10.1002/adhm.201400029. PMC4276556 24958085

[44] Liu AC, Joag VR, and Gotlieb AI (2007). The Emerging Role of Valve Interstitial Cell Phenotypes in Regulating Heart Valve Pathobiology. Am. J. Pathol. 171, 1407–1418. 10.2353/ajpath.2007.070251. 17823281 PMC2043503

[45] Schroeder ME, Batan D, Gonzalez Rodriguez A, Speckl KF, Peters DK, Kirkpatrick BE, Hach GK, Walker CJ, Grim JC, Aguado BA, (2023). Osteopontin activity modulates sex-specific calcification in engineered valve tissue mimics. Bioeng. Transl. Med. 8, e10358. 10.1002/btm2.10358. 36684107 PMC9842038

[46] Gorashi RM, Baddour T, Chittle SJ, Félix Vélez NE, Wenning MA, Anseth KS, Mestroni L, Peña B, Guo P, and Aguado BA (2025). Y chromosome–linked UTY modulates sex differences in valvular fibroblast methylation in response to nanoscale extracellular matrix cues. Sci. Adv. 11, eads5717. 10.1126/sciadv.ads5717. 40073144 PMC11900877

[47] Vogt BJ, Wang P, Chavez M, Guo P, Chow EK-H, Ho D, and Aguado BA (2025). Determining sex differences in aortic valve myofibroblast responses to drug combinations identified using a digital medicine platform. Sci. Adv. 11, eadu2695. 10.1126/sciadv.adu2695. 40479052 PMC12143354

[48] Lin R, Zhu Y, Chen W, Wang Z, Wang Y, and Du J (2024). Identification of Circulating Inflammatory Proteins Associated with Calcific Aortic Valve Stenosis by Multiplex Analysis. Cardiovasc. Toxicol. 24, 499–512. 10.1007/s12012-024-09854-5. 38589550

[49] Vogt BJ, Peters DK, Anseth KS, and Aguado BA (2022). Inflammatory serum factors from aortic valve stenosis patients modulate sex differences in valvular myofibroblast activation and osteoblast-like differentiation. Biomater. Sci. 10, 6341–6353. 10.1039/D2BM00844K. 36226463 PMC9741081

[50] Martin-Rojas T, Mourino-Alvarez L, Alonso-Orgaz S, Rosello-Lleti E, Calvo E, Lopez-Almodovar LF, Rivera M, Padial LR, Lopez JA, de la Cuesta F, and Barderas MG (2015). iTRAQ proteomic analysis of extracellular matrix remodeling in aortic valve disease. Sci. Rep. 5, 17290. 10.1038/srep17290. 26620461 PMC4664895

[51] Aguado BA, Schuetze KB, Grim JC, Walker CJ, Cox AC, Ceccato TL, Tan A-C, Sucharov CC, Leinwand LA, Taylor MRG, (2019). Transcatheter aortic valve replacements alter circulating serum factors to mediate myofibroblast deactivation. Sci. Transl. Med. 11, eaav3233. 10.1126/scitranslmed.aav3233. 31511425 PMC6754739

[52] Clift CL, Blaser MC, Bartoli-Leonard F, Schlotter F, Higashi H, Kuraoka S, Kasai T, Turner ME, Pham T, Perez KA, (2025). Sexual Dimorphism of Plasma and Tissue Proteomes in Human Calcific Aortic Valve Stenosis Pathogenesis. Arterioscler. Thromb. Vasc. Biol. 45, 1928–1934, 0. 10.1161/ATVBAHA.125.322560. 40808650

[53] Rogers JD, Aguado BA, Watts KM, Anseth KS, and Richardson WJ (2022). Network modeling predicts personalized gene expression and drug responses in valve myofibroblasts cultured with patient sera. Proc. Natl. Acad. Sci. USA 119, e2117323119. 10.1073/pnas.2117323119. 35181609 PMC8872767

[54] Keeney TR, Bock C, Gold L, Kraemer S, Lollo B, Nikrad M, Stanton M, Stewart A, Vaught JD, and Walker JJ (2009). Automation of the SomaLogic Proteomics Assay: A Platform for Biomarker Discovery. JALA: J. Assoc. Lab. Autom. 14, 360–366. 10.1016/j.jala.2009.05.003.

[55] Kraemer S, Schneider DJ, Paterson C, Perry D, Westacott MJ, Hagar Y, Katilius E, Lynch S, Russell TM, Johnson T, (2024). Crossing the Halfway Point: Aptamer-Based, Highly Multiplexed Assay for the Assessment of the Proteome. J. Proteome Res. 23, 4771–4788. 10.1021/acs.jproteome.4c00411. 39038188 PMC11536431

[56] Shelbaya K, Arthur V, Yang Y, Dorbala P, Buckley L, Claggett B, Skali H, Dufresne L, Yang T-Y, Engert JC, (2024). Large-Scale Proteomics Identifies Novel Biomarkers and Circulating Risk Factors for Aortic Stenosis. J. Am. Coll. Cardiol. 83, 577–591. 10.1016/j.jacc.2023.11.021. 38296402 PMC12582517

[57] Fairbanks BD, Schwartz MP, Halevi AE, Nuttelman CR, Bowman CN, and Anseth KS (2009). A Versatile Synthetic Extracellular Matrix Mimic via Thiol-Norbornene Photopolymerization. Adv. Mater. 21, 5005–5010. 10.1002/adma.200901808. 25377720 PMC4226179

[58] Feng N, Zhang Z, Wang Z, Zheng H, Qu F, He X, and Wang C (2015). Insulin-Like Growth Factor Binding Protein-2 Promotes Adhesion of Endothelial Progenitor Cells to Endothelial Cells via Integrin α5β1. J. Mol. Neurosci. 57, 426–434. 10.1007/s12031-015-0589-3. 26076738

[59] Schütt BS, Langkamp M, Rauschnabel U, Ranke MB, and Elmlinger MW (2004). Integrin-mediated action of insulin-like growth factor binding protein-2 in tumor cells. J. Mol. Endocrinol. 32, 859–868. 10.1677/jme.0.0320859. 15171717

[60] Lew J, Sanghavi M, Ayers CR, McGuire DK, Omland T, Atzler D, Gore MO, Neeland I, Berry JD, Khera A, (2017). Sex-Based Differences in Cardiometabolic Biomarkers. Circulation 135, 544–555. 10.1161/CIRCULATIONAHA.116.023005. 28153991 PMC5302552

[61] Raafs A, Verdonschot J, Ferreira JP, Wang P, Collier T, Henkens M, Björkman J, Boccanelli A, Clark AL, Delles C, (2021). Identification of sex-specific biomarkers predicting new-onset heart failure. ESC Heart Fail. 8, 3512–3520. 10.1002/ehf2.13476. 34156155 PMC8497379

[62] Daniels LB, and Maisel AS (2015). Cardiovascular biomarkers and sex: the case for women. Nat. Rev. Cardiol. 12, 588–596. 10.1038/nrcardio.2015.105. 26149486

[63] Chang Z, Lu J, Zhang Q, Wu H, Liang Z, Pan X, Li B, Cheng ZJ, and Sun B (2024). Clinical biomarker profiles reveals gender differences and mortality factors in sepsis. Front. Immunol. 15, 1413729. 10.3389/fimmu.2024.1413729. 38835774 PMC11148215

[64] Younes S. (2024). The relationship between gender and pharmacology. Curr. Res. Pharmacol. Drug Discov. 7, 100192. 10.1016/j.crphar.2024.100192. 39101002 PMC11295939

[65] Aljohmani A, and Yildiz D (2026). Biological sex differences in pharmacokinetics and adverse drug reactions. Naunyn-Schmiedebergs Arch Pharmacol 399, 3285–3301. 10.1007/s00210-025-04721-8. 41117965 PMC12935741

[66] Faust MN, Nguyen AK, Gorashi RM, Vélez NE, Loud MC, and Aguado BA (2026). Transforming Growth Factor β1 Modulates Sex Differences in Cardiac Myofibroblast Activation on Hydrogel Biomaterials. Cell. Mol. Bioeng. 10.1007/s12195-026-00923-z.

[67] Varennes O, Mary A, Bricca G, Kamel S, and Bellien J (2019). Dipeptidyl Peptidase-4 Inhibition Prevents Vascular Calcification by Potentiating the Insulin-Like Growth Factor-1 Signaling Pathway. JACC, Basic Transl. Sci. 4, 113–115. 10.1016/j.jacbts.2018.11.002. 30847425 PMC6390676

[68] Ock S, Ham W, Kang CW, Kang H, Lee WS, and Kim J (2021). IGF-1 protects against angiotensin II-induced cardiac fibrosis by targeting αSMA. Cell Death Dis. 12, 688. 10.1038/s41419-021-03965-5. 34244467 PMC8270920

[69] Bach LA (2018). 40 YEARS OF IGF1: IGF-binding proteins. J. Mol. Endocrinol. 61, T11–T28. 10.1530/JME-17-0254. 29255001

[70] Friedrich N, Wolthers OD, Arafat AM, Emeny RT, Spranger J, Roswall J, Kratzsch J, Grabe HJ, Hübener C, Pfeiffer AFH, (2014). Age- and Sex-Specific Reference Intervals Across Life Span for Insulin-Like Growth Factor Binding Protein 3 (IGFBP-3) and the IGF-I to IGFBP-3 Ratio Measured by New Automated Chemiluminescence Assays. J. Clin. Endocrinol. Metab. 99, 1675–1686. 10.1210/jc.2013-3060. 24483154

[71] Lin C-M, Huang Y-L, and Lin Z-Y (2009). Influence of Gender on Serum Growth Hormone, Insulin-Like Growth Factor-I and Its Binding Protein-3 during Aging. Yonsei Med. J. 50, 407–413. 10.3349/ymj.2009.50.3.407. 19568604 PMC2703765

[72] Baxter RC (2023). Signaling Pathways of the Insulin-like Growth Factor Binding Proteins. Endocr. Rev. 44, 753–778. 10.1210/endrev/bnad008. 36974712 PMC10502586

[73] Tian F, Wang Y, and Bikle DD (2018). IGF-1 signaling mediated cell-specific skeletal mechano-transduction. J. Orthop. Res. 36, 576–583. 10.1002/jor.23767. 28980721 PMC5839951

[74] Muessig JM, Lichtenauer M, Wernly B, Kelm M, Franz M, Bäz L, Schulze PC, Racher V, Zimmermann G, Figulla H-R, (2019). Insulin like growth factor binding protein 2 (IGFBP-2) for risk prediction in patients with severe aortic stenosis undergoing Transcatheter Aortic Valve Implantation (TAVI). Int. J. Cardiol. 277, 54–59. 10.1016/j.ijcard.2018.09.091. 30309683

[75] Habeb M, Embarak S, Fathy A, and Zalat M (2021). Non-invasive IGFBP1, IGFBP2 biomarkers as predictors and outcomes of usual interstitial pneumonia (UIP) therapeutic response. Egypt. J. Bronchol. 15, 6. 10.1186/s43168-020-00050-x.

[76] Han S, Li Z, Master LM, Master ZW, and Wu A (2014). Exogenous IGFBP-2 promotes proliferation, invasion, and chemoresistance to temozolomide in glioma cells via the integrin β1-ERK pathway. Br. J. Cancer 111, 1400–1409. 10.1038/bjc.2014.435. 25093489 PMC4183856

[77] Lu H, Wang L, Gao W, Meng J, Dai B, Wu S, Minna J, Roth JA, Hofstetter WL, Swisher SG, and Fang B (2013). IGFBP2/FAK Pathway Is Causally Associated with Dasatinib Resistance in Non–Small Cell Lung Cancer Cells. Mol. Cancer Therapeut. 12, 2864–2873. 10.1158/1535-7163.MCT-13-0233. PMC3858413 24130049

[78] Schuermans A, Pournamdari AB, Lee J, Bhukar R, Ganesh S, Darosa N, Small AM, Yu Z, Hornsby W, Koyama S, (2024). Integrative proteomic analyses across common cardiac diseases yield mechanistic insights and enhanced prediction. Nat. Cardiovasc. Res. 3, 1516–1530. 10.1038/s44161-024-00567-0. 39572695 PMC11634769

[79] Lau ES, Binek A, Parker SJ, Shah SH, Zanni MV, Van Eyk JE, and Ho JE (2022). Sexual Dimorphism in Cardiovascular Biomarkers: Clinical and Research Implications. Circ. Res. 130, 578–592. 10.1161/CIRCRESAHA.121.319916. 35175850 PMC8883873

[80] Cai Z, Zhu M, Xu L, Wang Y, Xu Y, Yim WY, Cao H, Guo R, Qiu X, He X, (2024). Directed Differentiation of Human Induced Pluripotent Stem Cells to Heart Valve Cells. Circulation 149, 1435–1456. 10.1161/CIRCULATIONAHA.123.065143. 38357822 PMC11062615

[81] Huang Y, Shan Y, Zhang W, Lee AM, Li F, Stranger BE, and Huang RS (2023). Deciphering genetic causes for sex differences in human health through drug metabolism and transporter genes. Nat. Commun. 14, 175. 10.1038/s41467-023-35808-6. 36635277 PMC9837057

[82] Moyer AM, Matey ET, and Miller VM (2019). Individualized medicine: Sex, hormones, genetics, and adverse drug reactions. Pharmacol. Res. Perspect. 7, e00541. 10.1002/prp2.541. 31844524 PMC6897337

[83] Captur G, Doykov I, Chung S-C, Field E, Barnes A, Zhang E, Heenan I, Norrish G, Moon JC, Elliott PM, (2024). Novel Multiplexed Plasma Biomarker Panel Has Diagnostic and Prognostic Potential in Children With Hypertrophic Cardiomyopathy. Circ. Genom. Precis. Med. 17, e004448. 10.1161/CIRCGEN.123.004448. 38847081 PMC11188636

[84] Verschuren L, Mak AL, van Koppen A, Özsezen S, Difrancesco S, Caspers MPM, Snabel J, van der Meer D, van Dijk A-M, Rashu EB, (2024). Development of a novel non-invasive biomarker panel for hepatic fibrosis in MASLD. Nat. Commun. 15, 4564. 10.1038/s41467-024-48956-0. 38811591 PMC11137090

[85] Tseng H, Balaoing LR, Grigoryan B, Raphael RM, Killian TC, Souza GR, and Grande-Allen KJ (2014). A three-dimensional co-culture model of the aortic valve using magnetic levitation. Acta Biomater. 10, 173–182. 10.1016/j.actbio.2013.09.003. 24036238 PMC10593146

[86] Johnson CM, Hanson MN, and Helgeson SC (1987). Porcine cardiac valvular subendothelial cells in culture: Cell isolation and growth characteristics. J. Mol. Cell. Cardiol. 19, 1185–1193. 10.1016/S0022-2828(87)80529-1. 3327949

[87] Sudi S, Suresh SD, Kolli T, Azamar KM, Dickson A, Mohammadalizadeh Z, and Porras AM (2025). Trimethylamine-N-oxide promotes fibrotic activation of quiescent valvular interstitial cells via endoplasmic reticulum stress. Sci. Rep. 16, 2269. 10.1038/s41598-025-32038-2. 41453954 PMC12816747

