# Supplementary material for: Aortic valve stenosis serum IGFBP2 mediates female-specific Evogliptin resistance in valve myofibroblasts
Source: Cell Rep. Author manuscript; Available in PMC 2026 Aug 14. (PMC13476089; doi:10.1016/j.celrep.2026.117678)
Supplement: Supplementary file 1 [file NIHMS2199968-supplement-1.pdf]

Cell Reports, Volume *45*

Supplemental information

Aortic valve stenosis serum IGFBP2 mediates female-specific Evogliptin resistance in valve myofibroblasts

Brandon J. Vogt, Megan Chavez, Nicole E. Félix Vélez, Rayyan M. Gorashi, Ryan R. Reeves, and Brian A. Aguado

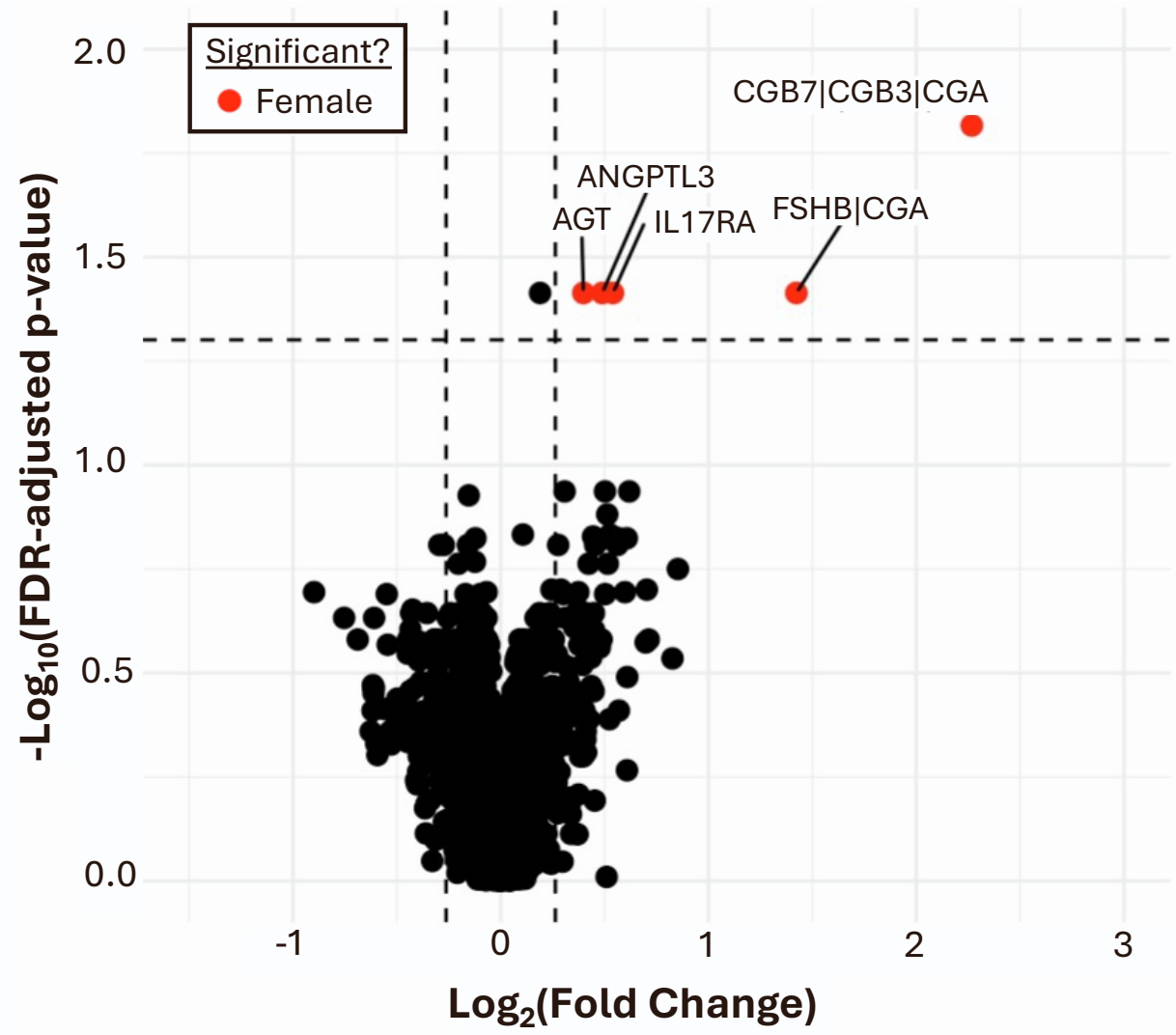

Supplemental Figure 1. Male and female AVS patient serum proteomes are similar. Volcano plot showing proteins that are more abundant in female AVS serum samples (red) (N=1,512 proteins, N=25 male serum samples, N=17 female serum samples). Statistical significance was defined as FDR-adjusted P-value < 0.05 (unpaired two-tailed t-test with Welch's correction) and fold change greater than 1.2.

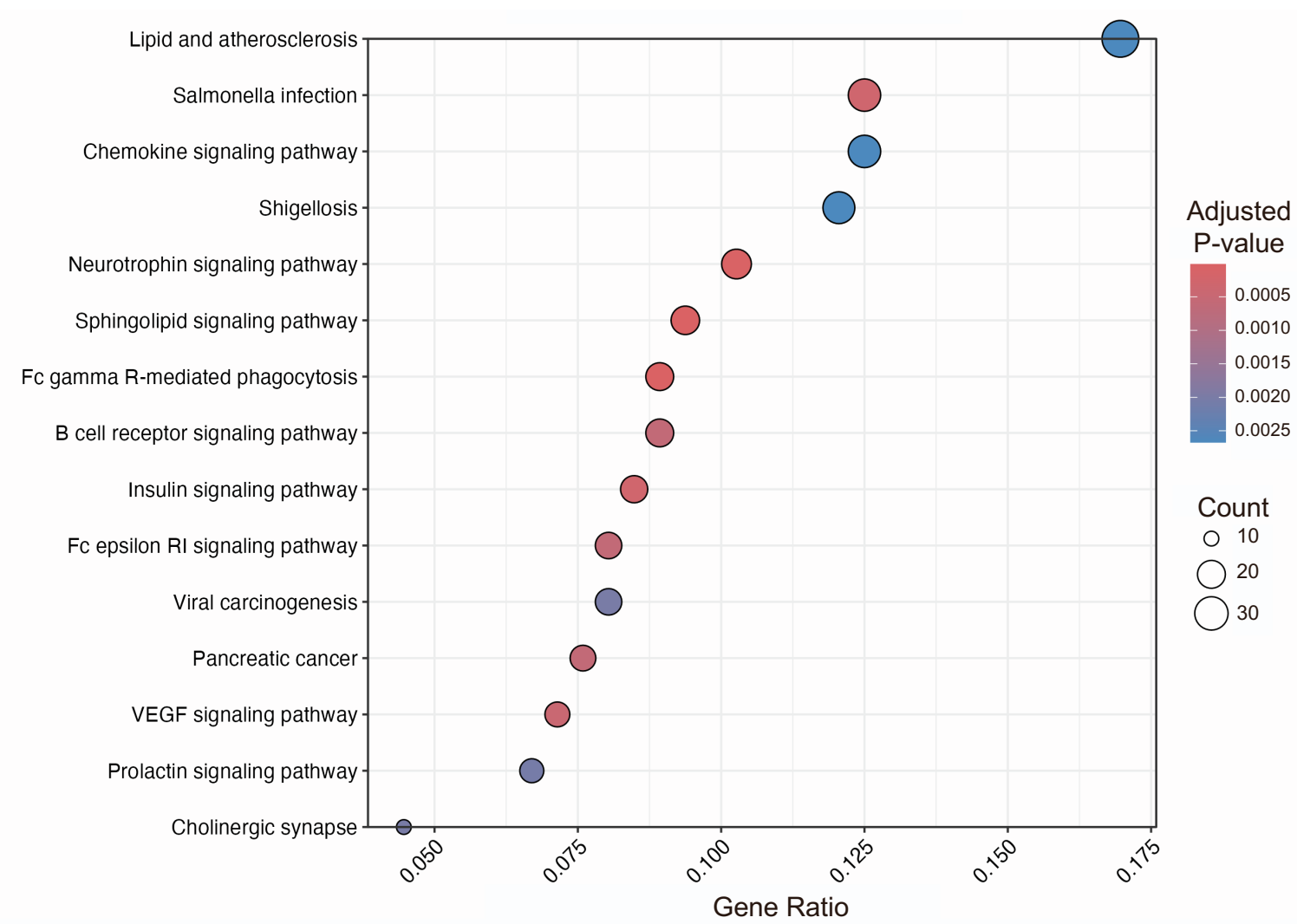

Supplemental Figure 2. Top enriched pathways identified from AVS serum samples. Top 15 pathways identified through KEGG analysis from the proteins that are more abundant in AVS serum samples.

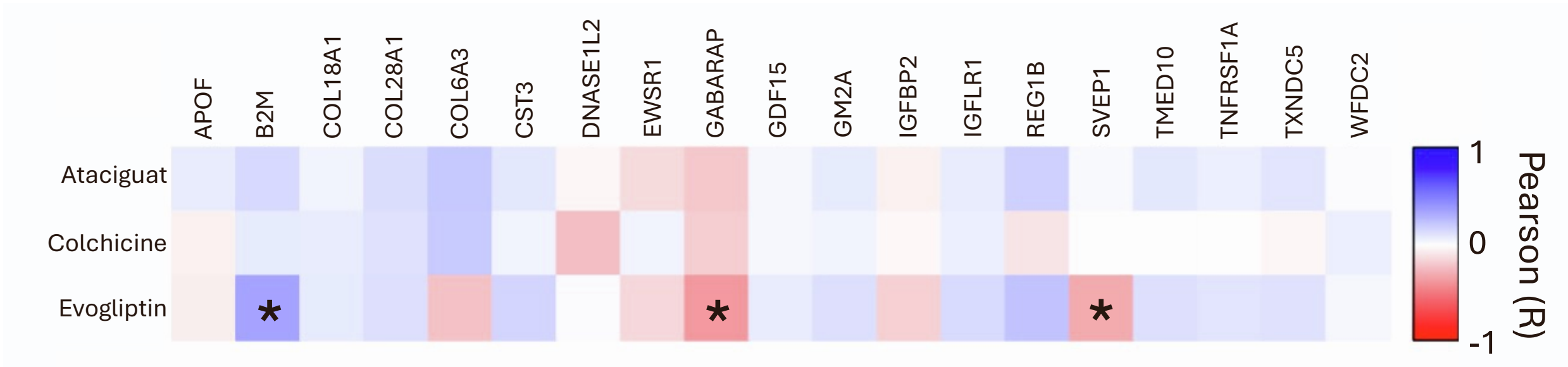

Supplemental Figure 3. Overall serum factor-drug correlations. Heatmap showing the overall correlation between each candidate serum factor and antifibrotic drug (N=31 serum samples). Statistical significance is indicated as * = P < 0.05.

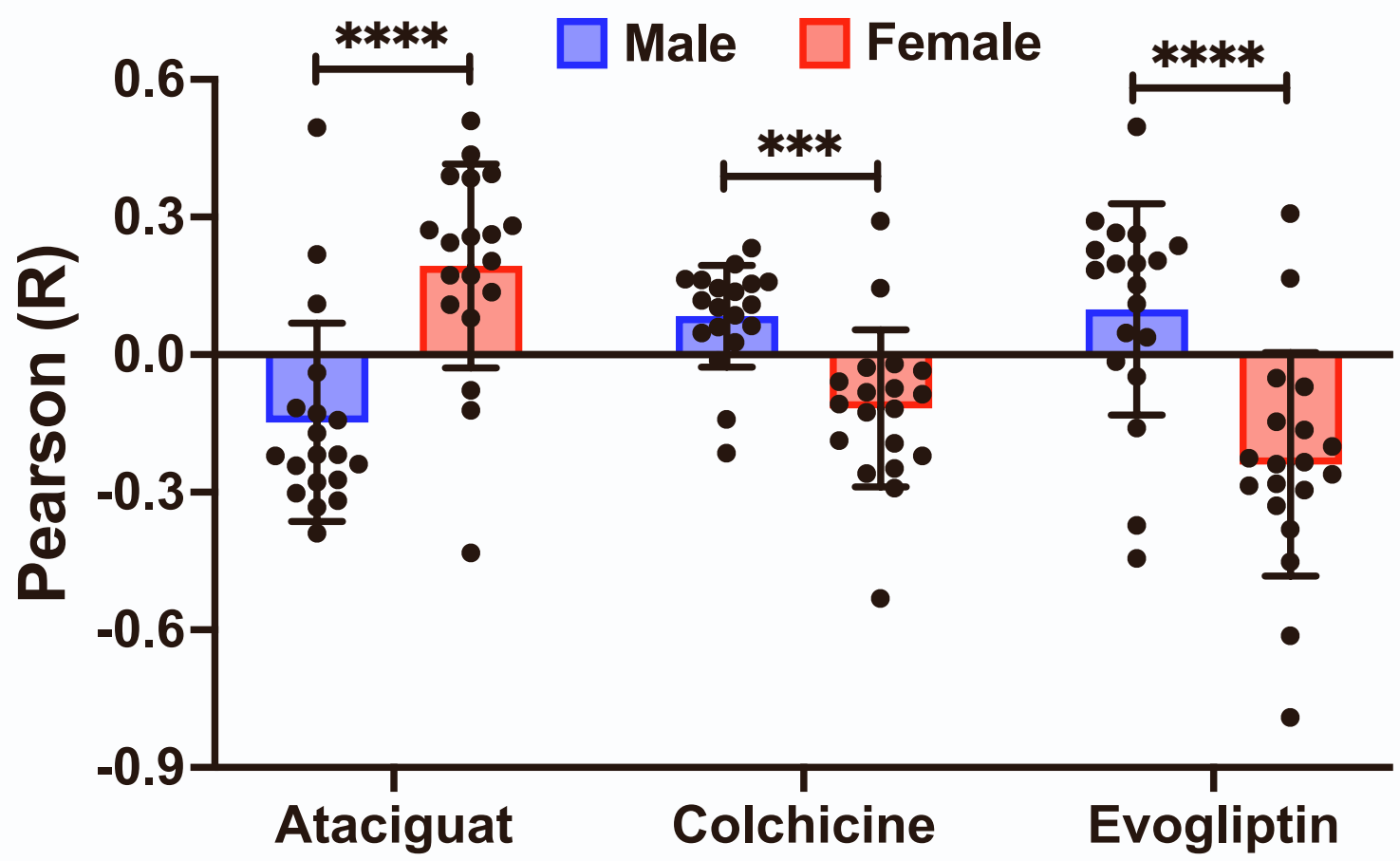

Supplemental Figure 4. Serum biomarker correlations with antifibrotic drugs are sex-specific. Pearson correlation of male and female serum biomarkers with Ataciguat, Colchicine, or Evogliptin efficacy in male and female VICs cultured on hydrogels with sex-matched AVS serum (N=19 serum biomarkers). Statistical significance is indicated as *** = P < 0.001; **** = P < 0.0001 (unpaired two-tailed t-test with Welch's correction).

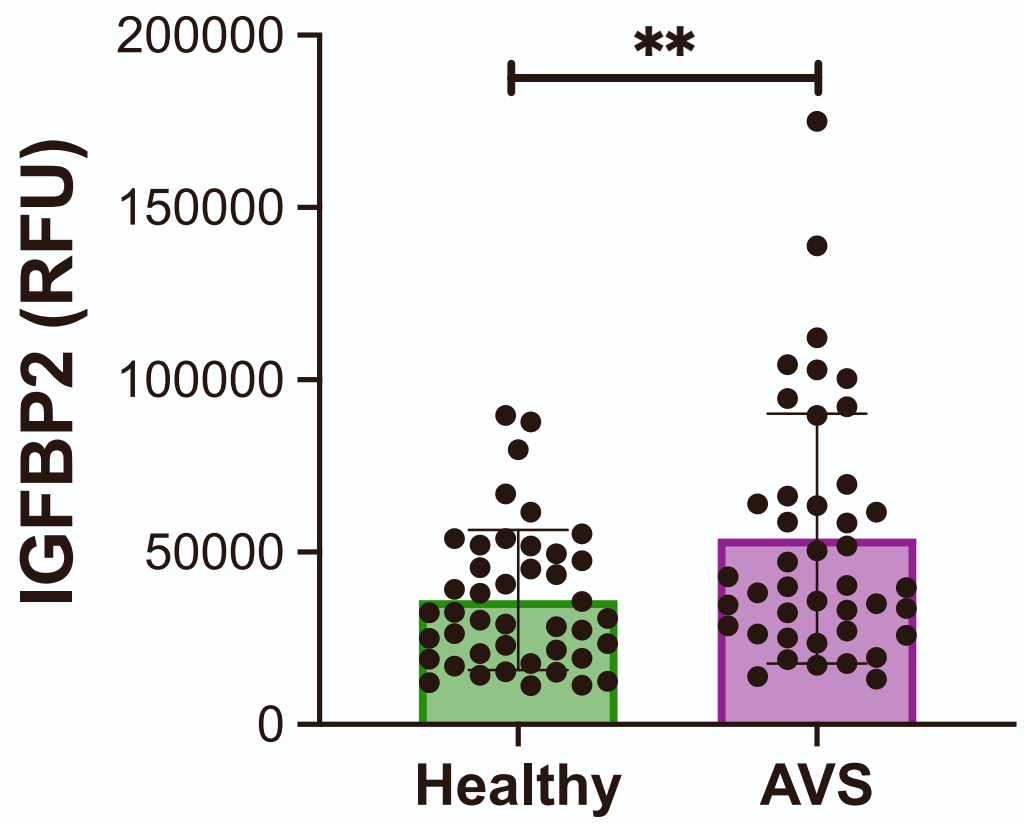

Supplemental Figure 5. IGFBP2 is more abundant in AVS patient serum samples. Serum IGFBP2 abundance in AVS patient and healthy serum samples quantified using relative fluorescent units (RFU) (N=42 AVS, N=43 healthy). Statistical significance is defined as P-value < 0.01 (unpaired two-tailed t-test with Welch's correction).

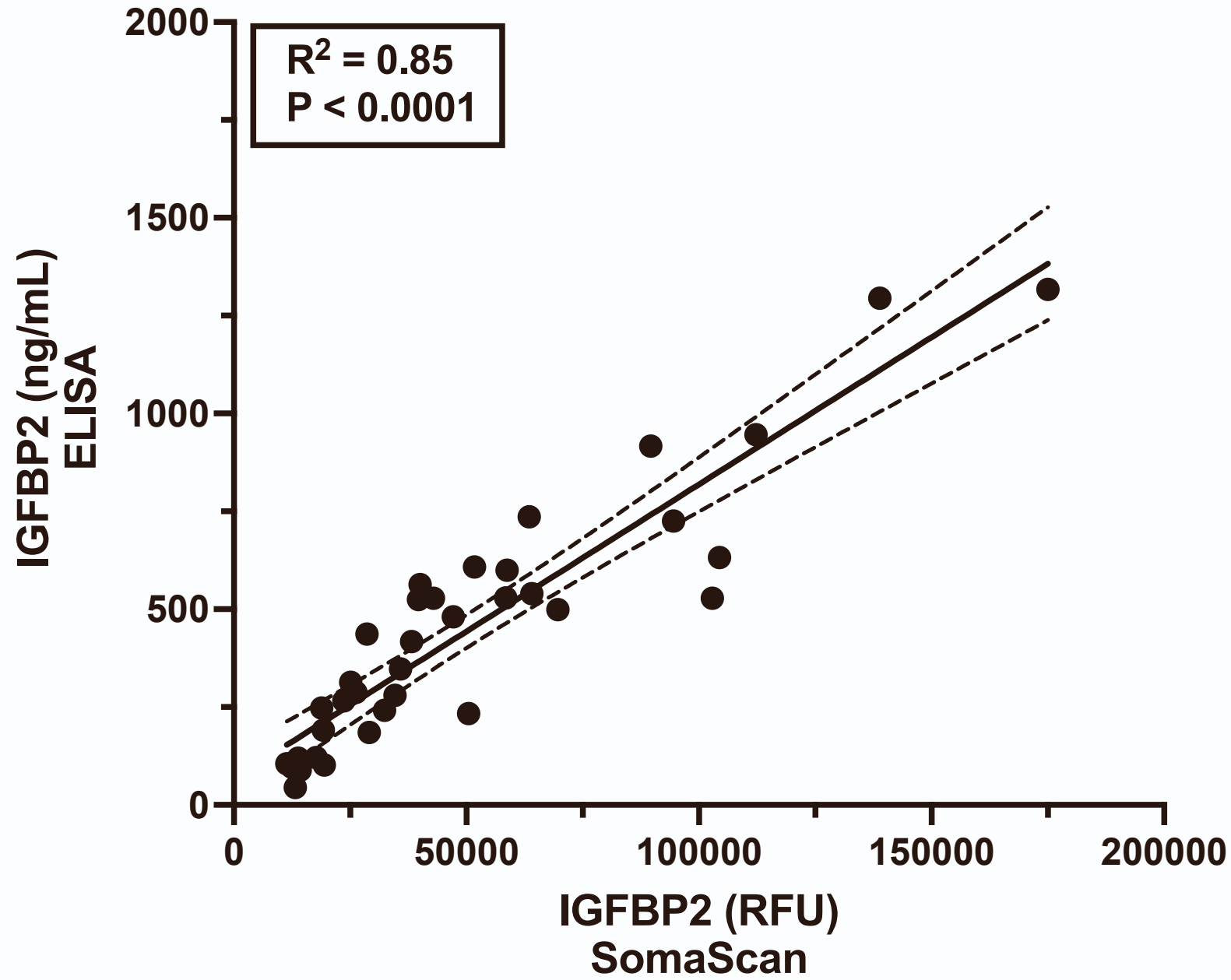

Supplemental Figure 6. IGFBP2 serum concentration is strongly correlated with IGFBP2 serum abundance. Correlation plot comparing AVS patient serum IGFBP2 abundance quantified from the SomaScan array and serum IGFBP2 concentration quantified from an ELISA kit (N=36 serum samples).

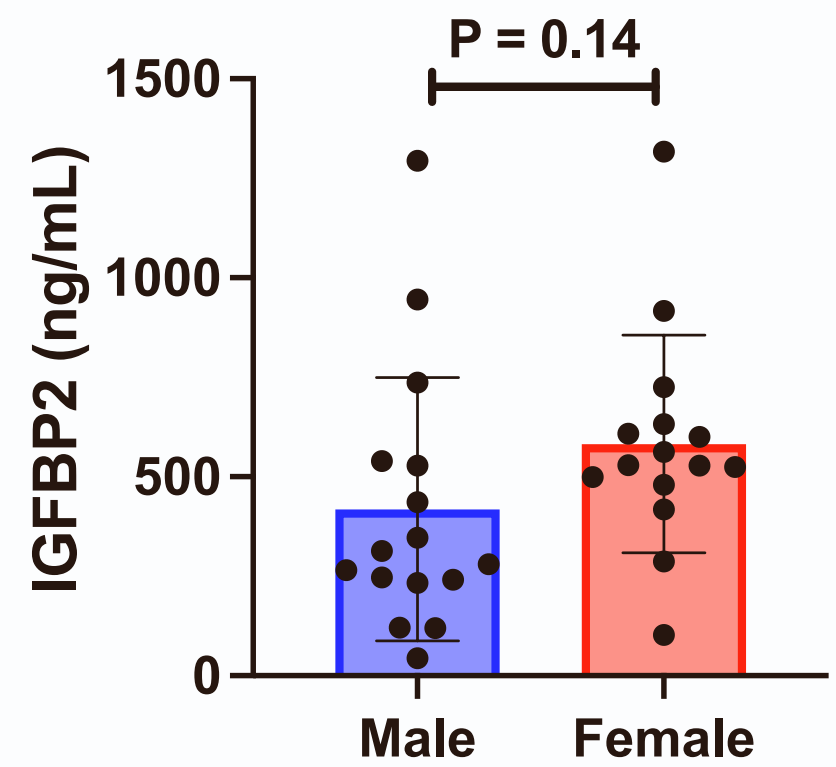

Supplemental Figure 7. Serum IGFBP2 concentration is not sex dependent. Bar graph comparing IGFBP2 concentrations in male and female AVS serum samples (N=16 male, N=15 female). Statistical significance was determined by unpaired two-tailed t-test with Welch's correction.

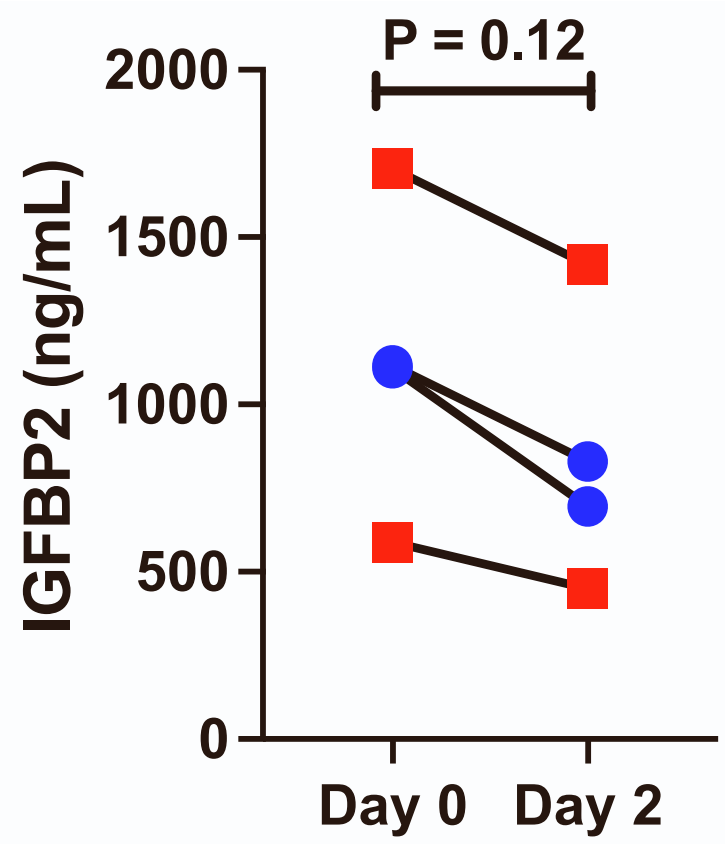

Supplemental Figure 8. IGFBP2 degradation in cell culture is minimal. Serum IGFBP2 levels after 0 days and 2 days of culture with sex-matched male (blue) and female (red) VICs on hydrogels (N=2 serum samples per sex). Statistical significance was determined by Wilcoxon test.

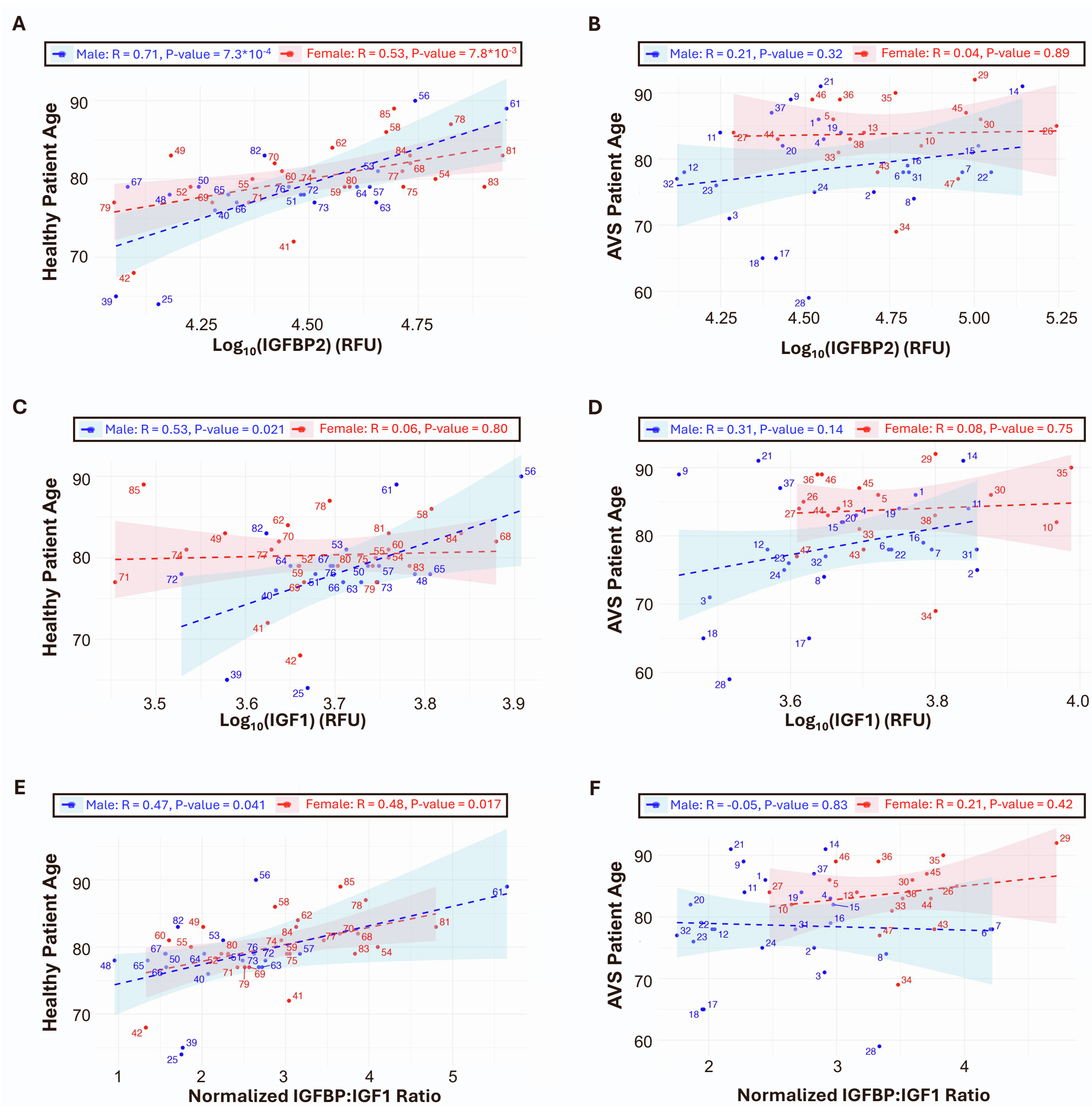

Supplemental Figure 9. Age correlates with serum IGFBP2, IGF1, and their ratios only in healthy patients. (A-B) Correlation between age and serum IGFBP2 abundance in male and female **(A)** healthy or **(B)** AVS patients. **(C-D)** Correlation between age and serum IGF1 abundance in male and female **(C)** healthy or **(D)** AVS patients. **(E-F)** Correlation between age and normalized IGFBP:IGF1 ratio in male and female **(E)** healthy or **(F)** AVS patients. (N=19 male healthy, N=24 female healthy, N=25 male AVS, N=17 female AVS).

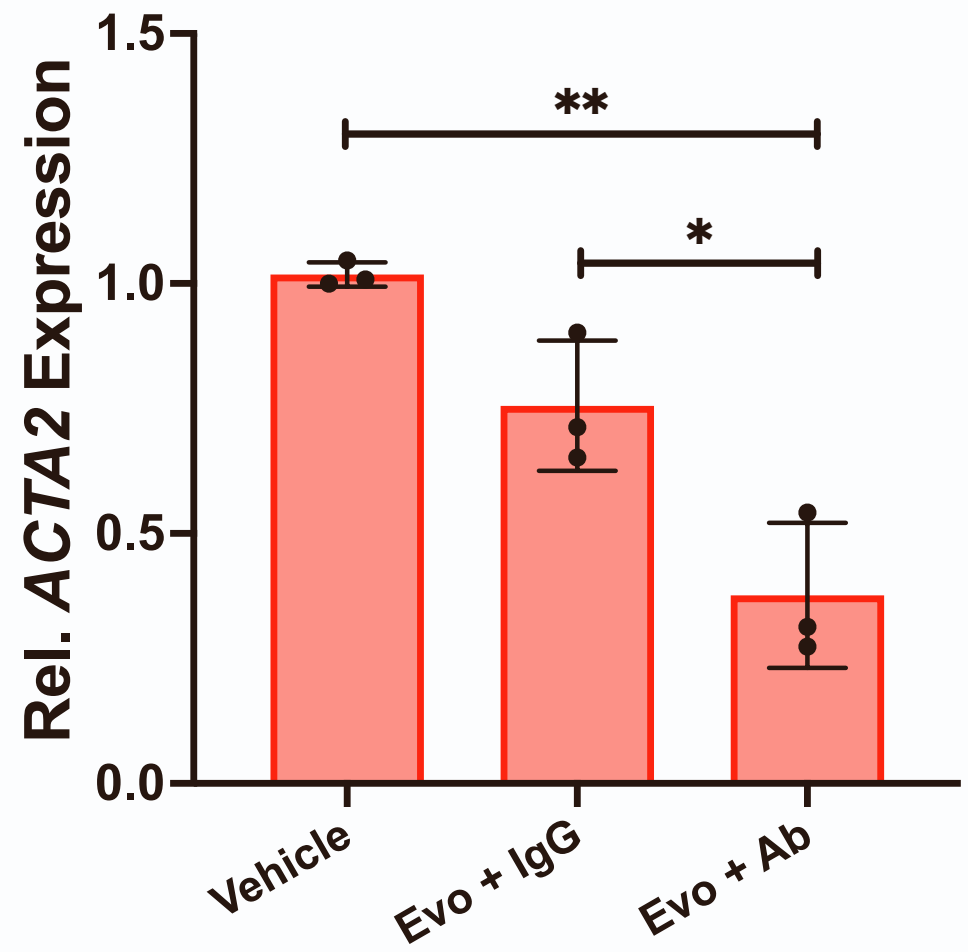

Supplemental Figure 10. *ACTA2* gene expression is more robustly reduced by combining Evogliptin and IGFBP2 neutralizing antibody treatment. RT-qPCR of *ACTA2* gene expression in vehicle, Evogliptin plus IgG antibody (Evo + IgG), and Evogliptin plus IGFBP2 neutralizing antibody (Evo + Ab) treated groups (N=3 serum samples). Statistical significance is indicated as * = P <0.05 and ** = P < 0.01 (one-way ANOVA with Tukey posttests).

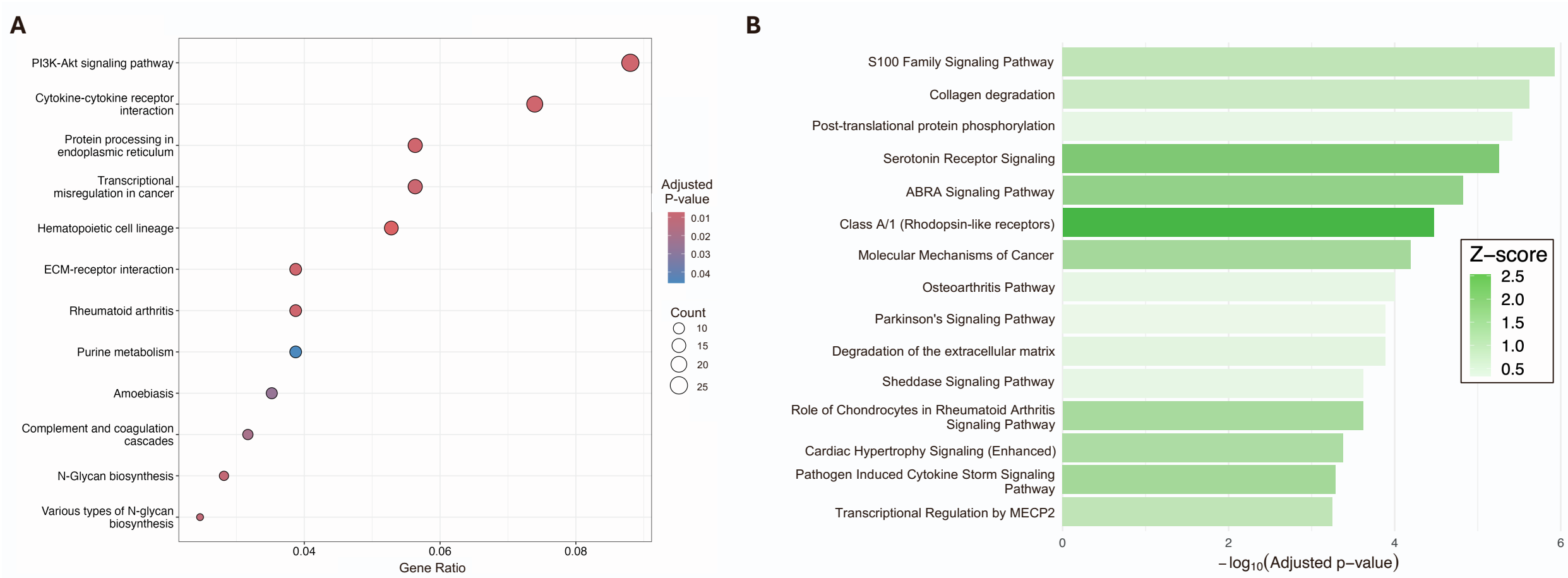

Supplemental Figure 11. Enriched pathways in female VICs treated with IGFBP2 neutralizing antibody and Evogliptin. (A-B) Top upregulated pathways in the Evogliptin plus IGFBP2 neutralizing antibody group versus the Evogliptin group, identified by **(A)** KEGG and **(B)** Ingenuity Pathway Analysis.

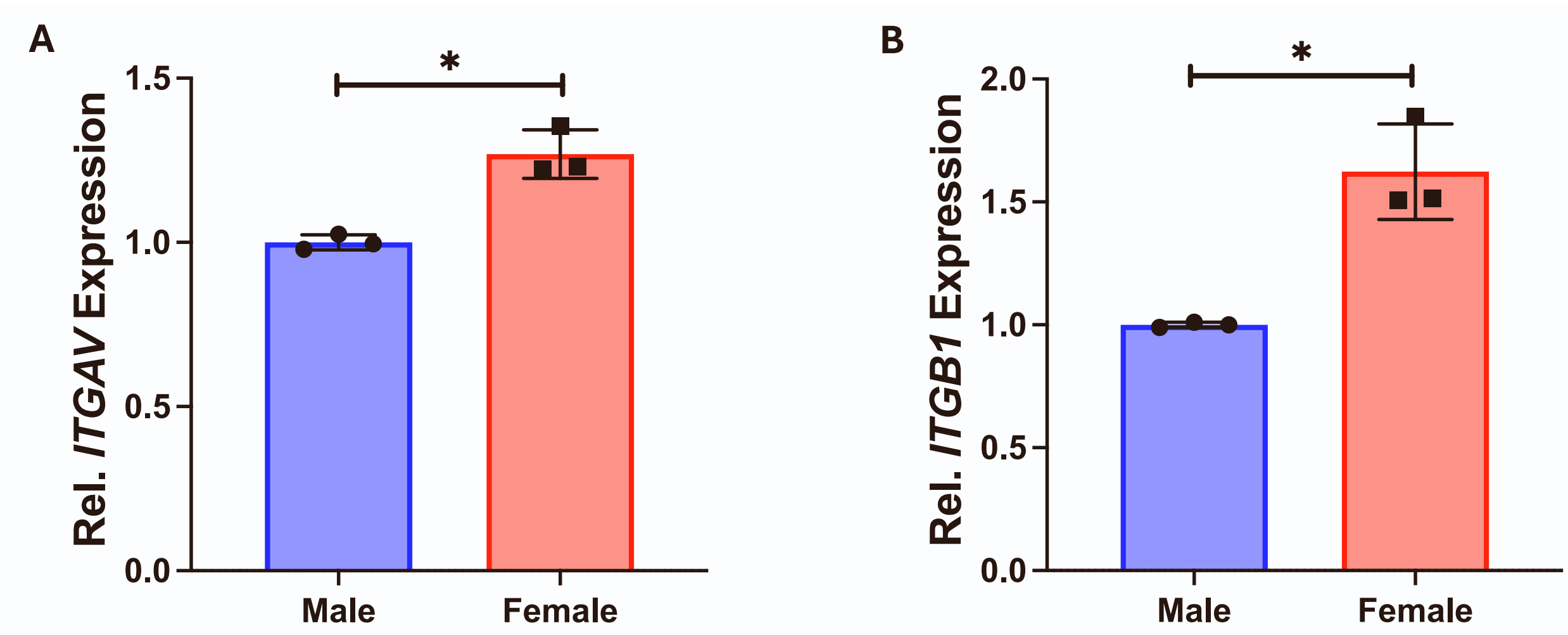

Supplemental Figure 12. Female VICs have increased *ITGAV* and *ITGB1* expression relative to male VICs. (A-B) RT-qPCR of **(A)** *ITGAV* and **(B)** *ITGB1* gene expression in male and female VICs cultured on hydrogels (N=3 biological replicates). Statistical significance is indicated as * = $P < 0.05$ (unpaired two-tailed t-test with Welch's correction).

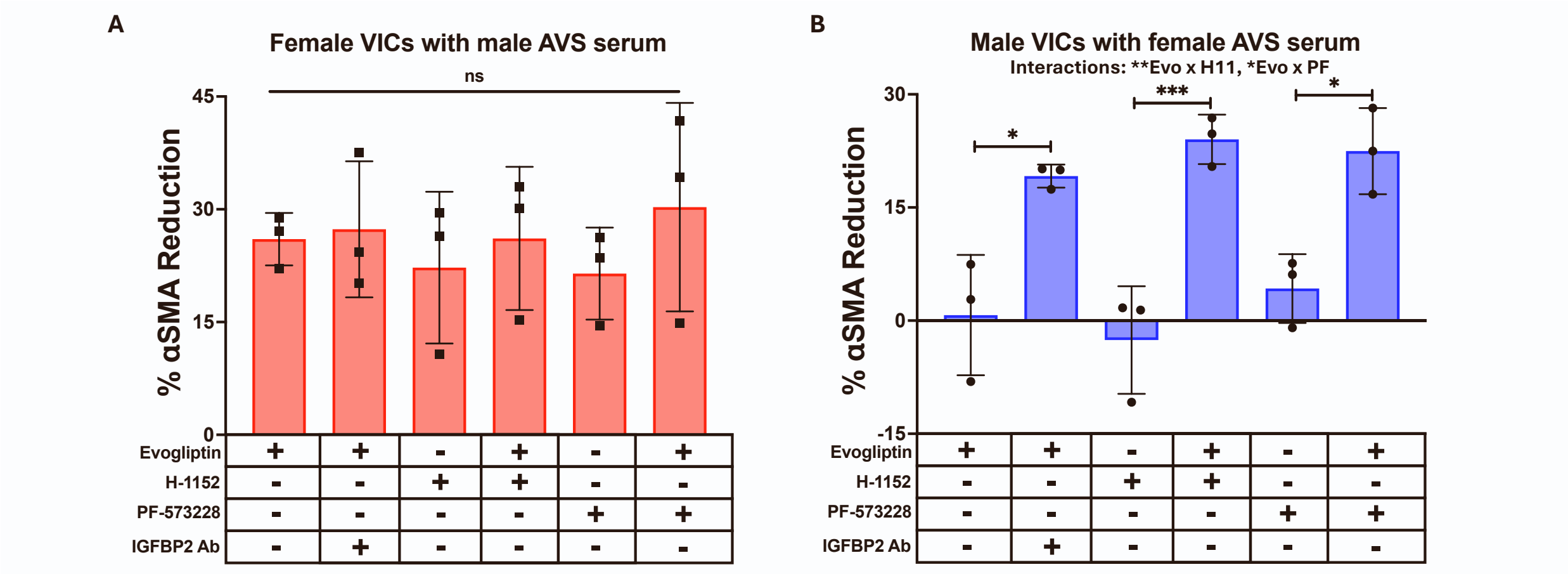

Supplemental Figure 13. Swapping serum for male and female VICs confirms that female AVS serum disrupts Evogliptin efficacy through Rho/ROCK and focal adhesion kinase signaling. (A-B) Percent αSMA reduction in **(A)** female or **(B)** male VICs cultured on hydrogels with opposite sex AVS serum and treated with various combinations of Evogliptin, IGFBP2 neutralizing antibody, H-1152 , and PF-573228 (N=3 hydrogels per sex). Statistical significance between groups is indicated as * = P < 0.05, *** = P < 0.01 and significant interactions between variables are indicated as * = P < 0.05, ** = P < 0.01 (two-way ANOVA with Tukey posttests).

Supplemental Table 1. Male and female AVS patient clinical data comparisons. Statistical significance was determined by unpaired t-test with Welch's correction. Bolded p-values are significant (P<0.05).

**Characteristic**	**Male Cohort (N = 25)**	**Female Cohort (N = 17)**	**P-value**
Age (years)	78 ± 8	84 ± 6	**0.016**
Aortic valve max velocity (m/s)	3.91 ± 0.97	4.00 ± 0.52	0.702
Aortic valve area (m^2)	1.15 ± 0.85	0.76 ± 0.18	**0.035**
Mean Pressure Gradient (mmHg)	37.4 ± 18.2	38.1 ± 9.0	0.867
SVI (mL/m^2)	52.4 ± 20.9	46.3 ± 14.1	0.270
LVIDD (cm)	4.44 ± 0.77	3.96 ± 0.79	0.063
LVIDS (cm)	2.85 ± 0.77	2.63 ± 0.77	0.364
Ejection fraction (%)	62.2 ± 11.0	64.4 ± 11.3	0.540
WBC ($1000/mm^2$)	7.39 ± 2.17	7.32 ± 1.87	0.911
Diastolic Volume (ml)	96.3 ± 33.2	77.4 ± 39.5	0.134
Systolic Volume (ml)	33.2 ± 13.4	35.9 ± 31.6	0.752
Aspirin (%)	60	53	0.661
Statin (%)	17	25	0.585
Losartan (%)	64	65	0.964

Supplemental Table 2. Clinically relevant dosing parameters for Ataciguat, Colchicine, and Evogliptin.

Drug	5% Cmax (μM)	10% Cmax (μM)	50% Cmax (μM)
Ataciguat	3.47	6.94	34.7
Colchicine	$7.76*10^{-4}$	$1.55*10^{-3}$	$7.76*10^{-3}$
Evogliptin	$2.70*10^{-3}$	$5.41*10^{-3}$	$2.70*10^{-2}$

Supplemental Table 3. Overall correlations (Pearson's R) between candidate serum biomarker abundance and percent αSMA reduction in male and female VICs cultured on hydrogels with sex-matched AVS serum and antifibrotic drugs (N=42 serum samples). Bolded correlations are significant (P<0.05).

Protein	Overall Correlation (Pearson's R) with Ataciguat	Overall Correlation (Pearson's R) with Colchicine	Overall Correlation (Pearson's R) with Evogliptin
APOF	0.072	-0.055	-0.066
B2M	0.153	0.085	**0.359**
COL18A1	0.045	0.080	0.081
COL28A1	0.132	0.111	0.125
COL6A3	0.220	0.210	-0.229
CST3	0.095	0.050	0.164
DNASE1L2	-0.037	-0.238	0.014
EWSR1	-0.137	0.043	-0.146
GABARAP	-0.208	-0.188	**-0.378**
GDF15	0.033	0.037	0.073
GM2A	0.082	0.040	0.129
IGFBP2	-0.054	-0.020	-0.177
IGFLR1	0.076	0.066	0.143
REG1B	0.181	-0.110	0.236
SVEP1	0.027	0.004	**-0.312**
TMED10	0.093	0.004	0.123
TNFRSF1A	0.062	-0.009	0.102
TXNDC5	0.106	-0.033	0.116
WFC2	0.019	0.069	0.032

Supplemental Table 4. Correlations (Pearson's R) between male candidate serum biomarker abundance and percent αSMA reduction in male VICs cultured on hydrogels with serum and antifibrotic drugs (N=25 serum samples). Bolded correlations are significant ($P<0.05$).

Protein	Male Correlation (Pearson's R) with Ataciguat	Male Correlation (Pearson's R) with Colchicine	Male Correlation (Pearson's R) with Evogliptin
APOF	-0.318	0.047	0.038
B2M	0.219	0.137	**0.497**
COL18A1	-0.273	0.154	0.205
COL28A1	-0.039	0.232	0.228
COL6A3	**0.495**	0.165	-0.160
CST3	-0.218	0.145	0.266
DNASE1L2	0.111	-0.141	-0.048
EWSR1	-0.302	0.197	0.047
GABARAP	-0.171	-0.215	-0.372
GDF15	-0.218	0.103	0.198
GM2A	-0.242	0.086	0.199
IGFBP2	-0.333	0.027	-0.015
IGFLR1	-0.239	0.159	0.262
REG1B	-0.128	-0.013	0.291
SVEP1	-0.221	0.109	**-0.444**
TMED10	-0.143	0.060	0.237
TNFRSF1A	-0.278	0.118	0.184
TXNDC5	-0.116	0.063	0.152
WFC2	-0.389	0.163	0.111

Supplemental Table 5. Correlations (Pearson's R) between female candidate serum biomarker abundance and percent αSMA reduction in female VICs cultured on hydrogels with serum and antifibrotic drugs (N=17 serum samples). Bolded correlations are significant (P<0.05).

Protein	Female Correlation (Pearson's R) with Ataciguat	Female Correlation (Pearson's R) with Colchicine	Female Correlation (Pearson's R) with Evogliptin
APOF	0.385	-0.193	-0.329
B2M	-0.432	0.145	0.166
COL18A1	0.136	-0.020	**-0.613**
COL28A1	0.262	-0.126	-0.226
COL6A3	-0.078	0.291	-0.381
CST3	0.173	-0.086	-0.261
DNASE1L2	0.272	**-0.531**	0.308
EWSR1	0.257	-0.259	-0.452
GABARAP	0.109	-0.221	-0.295
GDF15	0.080	-0.035	-0.281
GM2A	**0.510**	-0.028	-0.146
IGFBP2	-0.121	-0.059	**-0.791**
IGFLR1	0.436	-0.108	-0.234
REG1B	0.244	-0.291	-0.070
SVEP1	0.172	-0.118	-0.200
TMED10	0.281	-0.073	-0.239
TNFRSF1A	0.394	-0.248	-0.164
TXNDC5	0.204	-0.187	-0.051
WFC2	0.390	-0.082	-0.286

Supplemental Table 6. Forward and reverse primer sequences for genes assessed via RT-qPCR. All primers are shown from the 5' to 3' end.

Gene	Forward Primer (5'-3')	Reverse Primer (5'-3')
RPL30	AGATTTCCTCAAGGCTGGGC	GCTGGGGTACAAGCAGACTC
ACTA2	GCAAACAGGAATACGATGAAGCC	AACACATAGGTAACGAGTCAGAGC
ITGAV	ATGCAACAGGCAACAGAGACT	TCCTGAAGAAAGCAAGTTCCA
ITGB1	CAAGCAGGGCCAAATTGTGG	CCTTTGCTACGGTTGGTTACATT
